# Diet, Genes, or Both? Coffee Consumption, Snacking, and BDNF and TCF7L2 Genotypes in Relation to Cardiometabolic Traits: A Cross-Sectional Study in Polish Adults

**DOI:** 10.3390/nu18213545

**Published:** 2026-10-27

**Authors:** Witold Bauer, Filip Bossowski, Magdalena Bossowska, Natalia Momotko, Helena Żórawska, Jakub Targoński, Krzysztof Drygalski, Maria Górska, Adam Krętowski, Edyta Adamska-Patruno, Katarzyna Maliszewska

**Affiliations:** 1 Clinical Research Centre, Medical University of Białystok, M. Skłodowskiej-Curie 24A, 15-276 Białystok, Poland; 2 Department of Endocrinology, Diabetology and Internal Medicine, Medical University of Białystok, M. Skłodowskiej-Curie 24A, 15-276 Białystok, Poland; 3 Students’ Research Group, Department of Endocrinology, Diabetology and Internal Medicine, Medical University of Białystok, M. Skłodowskiej-Curie 24A, 15-276 Białystok, Poland; 4 Department of Hypertension and Diabetology, Medical University of Gdańsk, Mariana Smoluchowskiego 17, 80-214 Gdańsk, Poland; 5 Clinical Research Support Centre, Medical University of Białystok, M. Skłodowskiej-Curie 24A, 15-276 Białystok, Poland; 6 Department of Nutriomics, Clinical Research Centre, Medical University of Białystok, M. Skłodowskiej-Curie 24A, 15-276 Białystok, Poland

**Keywords:** nutrigenetics, gene–diet interaction, BDNF, TCF7L2, coffee consumption, snacking frequency, type 2 diabetes mellitus, oral glucose tolerance test

## Abstract

Background/Objectives: Coffee consumption and snacking are common dietary behaviours linked to cardiometabolic risk, and variants in BDNF and TCF7L2 have been associated with obesity and type 2 diabetes (T2DM). We examined whether glycaemic, adiposity, and lipid traits reflect these behaviours, these genotypes, or both, and whether BDNF (rs10501087) and TCF7L2 (rs4506565, rs7901695, rs7903146) genotypes modify the behavioural associations. Methods: In a cross-sectional study of Polish adults (NCT03792685; analytic cohort *n* = 1227), coffee consumption (never, weekly, daily) and snacking frequency (rare, frequent) were assessed by questionnaire (*n* = 905); outcomes were glucose and insulin area under the curve (AUC) from a 2-h 75 g oral glucose tolerance test (OGTT), glycated haemoglobin (HbA1c), fasting lipids, and bioimpedance body composition. Multivariable models were adjusted for age, sex, daily energy intake, and physical inactivity; all analyses, including gene × behaviour interaction tests, were exploratory, with false discovery rate (FDR) correction for multiple testing. Results: Daily coffee consumption was associated with a higher glucose AUC than never consumption (β = +0.045, 95% confidence interval [CI] 0.018–0.073; *p* = 0.001; FDR q = 0.094), with higher post-load glucose and insulin but similar fasting glucose and HbA1c; the association was robust in sensitivity analyses. Weekly coffee consumption was associated with higher high-density lipoprotein cholesterol (β = +4.75 mg/dL, 95% CI 1.49–8.02; *p* = 0.004; FDR q = 0.10). Frequent snacking was associated with higher body mass index, fat mass, and body fat percentage (nominal *p* = 0.034–0.049; FDR q = 0.26) and was not associated with coffee consumption. BDNF rs10501087 CT was nominally associated with lower T2DM odds than TT (odds ratio [OR] = 0.42, 95% CI 0.21–0.78; *p* = 0.009). The behavioural associations showed no detectable modification by genotype (184 interaction tests; none with FDR q < 0.05). Conclusions: Everyday dietary behaviour, particularly daily coffee consumption, was more consistently related to cardiometabolic traits than BDNF or TCF7L2 genotypes. The post-load glycaemic profile of daily coffee consumers, missed by fasting glucose and HbA1c, highlights the value of OGTT-based phenotyping in nutritional research. These exploratory findings do not support genotype-tailored advice on coffee or snacking.

## 1. Introduction

Coffee is among the most widely consumed beverages worldwide and is often drunk alongside meals or snacks. Its additives (e.g., sugar, milk/cream), preparation methods, and serving sizes vary and can alter the metabolic “dose” and interpretation of coffee as an exposure [1,2]. Such behavioural clustering is clinically relevant because it may confound or attenuate associations attributed to coffee itself, particularly when coffee is evaluated without consideration of the broader dietary pattern in which it is consumed [1].

Type 2 diabetes mellitus (T2DM) and related cardiometabolic phenotypes, including central adiposity, dyslipidaemia, and insulin resistance, are major contributors to morbidity and mortality. Prediabetes represents a high-risk but potentially modifiable metabolic state in which lifestyle exposures are particularly relevant [3]. However, the metabolic effects of lifestyle behaviours vary across individuals, and a uniform interpretation of common habits, including coffee consumption and snacking frequency, is often inadequate. A genetics-informed framework is therefore both biologically plausible and clinically relevant, as inherited susceptibility may influence baseline glycaemic risk and modify metabolic responses to lifestyle exposures [4,5].

In this context, two candidate gene pathways are of particular interest. The transcription factor 7-like 2 gene (TCF7L2) encodes a high mobility group (HMG)-box-containing transcription factor implicated in glucose homeostasis and insulin secretion [6,7], and common variants at this locus have been extensively associated with T2DM susceptibility [8,9]. Brain-derived neurotrophic factor (BDNF), a neurotrophin involved in neuronal signalling, contributes to appetite regulation and energy homeostasis, providing a plausible mechanistic link between eating behaviour, including snacking, and adiposity- and glycaemia-related traits [10,11]. Together, these loci support a nutrigenetic framework in which routine behaviours are considered not only as exposures but also as phenotypes that may interact with inherited susceptibility. Within this framework, we focused on common intronic variants that capture T2DM- and obesity-related signals at these loci in European populations, specifically rs4506565, rs7901695, and rs7903146 in TCF7L2 and rs10501087 in BDNF [9,12].

Consistent with this framework, TCF7L2 variants have been strongly associated with T2DM susceptibility [13] and altered glucose metabolism, including pathways related to insulin secretion and β-cell function [14,15] and insulin resistance [16]; TCF7L2 itself also regulates adipocyte development and function [17]. BDNF complements this pathway by linking appetite regulation, energy homeostasis, and glucose counter-regulation [18,19,20] with adiposity- and glycaemia-related phenotypes [21,22] and has been studied in relation to metabolic health and T2DM [23,24].

Against this mechanistic background, habitual coffee intake has been associated with metabolic outcomes, including a lower risk of metabolic syndrome and T2DM [2,25], although cross-sectional findings for individual components have been mixed [26]. However, coffee is a complex biochemical exposure [27] whose observed associations may be confounded by co-occurring behaviours such as smoking [28]. Self-reported coffee intake is commonly operationalised using broad frequency categories that do not capture brewing techniques, additives (e.g., sugar, milk), or co-occurring eating occasions, such as snacking [26,27,29]. Coffee–metabolic associations are therefore best evaluated within their broader behavioural context [30].

In a nutrigenetic framework, these considerations raise the broader question of whether cardiometabolic traits reflect diet, genes, or both, and two related questions in particular: whether TCF7L2 and BDNF variants are associated with glycaemic and adiposity phenotypes, and whether inherited susceptibility modifies associations between coffee and snacking behaviours and metabolic outcomes. Collectively, the existing literature indicates that coffee–metabolic associations are most informative when preparation context and co-occurring behaviours, including snacking, are considered [1,27]. It also supports incorporating genetic susceptibility to evaluate genotype–phenotype relationships and to determine whether genotype modifies behaviour–phenotype associations [4,5].

Accordingly, in a cross-sectional adult Polish cohort (project NCT03792685), we examined habitual coffee consumption and snacking frequency as behavioural phenotypes in relation to cardiometabolic markers of glycaemia, adiposity, and lipid metabolism, and evaluated whether these associations differed according to the BDNF variant rs10501087 and TCF7L2 variants (rs4506565, rs7901695, rs7903146). Specifically, we aimed to (i) characterise coffee- and snacking-related metabolic associations; (ii) assess genotype–phenotype relationships for BDNF rs10501087 and TCF7L2 variants; and (iii) explore genotype × behaviour interactions as a hypothesis-generating analysis.

## 2. Materials and Methods

This cross-sectional study was conducted as part of the ClinicalTrials.gov-registered project NCT03792685 and examined the associations of snacking frequency, coffee consumption, and genetic predisposition (BDNF and TCF7L2 polymorphisms) with glucose metabolism and metabolic risk. Participants were recruited by the Department of Endocrinology, Diabetology and Internal Medicine, Medical University of Białystok, Poland, and its affiliated Clinical Research Centre. Written informed consent was obtained from all participants before enrolment. The study was conducted in accordance with the Declaration of Helsinki and was approved by the Ethics Committee of the Medical University of Białystok, Poland (R-I-002/35/2009, approved on 29 January 2009).

The main inclusion criteria were: (1) age 18–79 years; (2) no major dietary or medication changes during the preceding 3 months; and (3) willingness to complete behavioural and dietary assessments. Individuals with major acute or chronic diseases other than T2DM, and those following weight-loss regimens or other interventions that could influence study outcomes, were excluded. The analytic cohort comprised 1227 participants with complete clinical and anthropometric assessments. Coffee consumption and snacking frequency questionnaires were completed by 905 participants (729 of whom belonged to the analytic cohort), who formed the analytic sample for all behavioural analyses; energy intake from 3-day dietary records was available for 464 of them and was used in the adjusted models (Figure S1).

### 2.1. Dietary and Behavioural Phenotyping

Behavioural exposures were assessed during the clinical visit using self-administered questionnaires. Habitual coffee consumption was evaluated using a dedicated questionnaire and categorised as never, weekly, or daily. Snacking behaviour, defined as consumption of food or caloric beverages between main meals, was assessed using a snacking frequency questionnaire. Participants were classified as rare snackers (<2 between-meal eating occasions/day) or frequent snackers (≥2 between-meal eating occasions/day).

To assess dietary covariates and energy intake, participants completed detailed 3-day food records documenting all meals, snacks, and beverages, together with portion sizes, using standard photographic aids. Total daily energy intake (kcal) and macronutrient composition were calculated using Dieta 6 software (National Food and Nutrition Institute, Warsaw, Poland).

Daily physical activity was evaluated using the self-administered International Physical Activity Questionnaire-Long Form (IPAQ-LF), a validated instrument that captures both duration and frequency of physical activities across different intensity levels, including walking, moderate exercise, and vigorous exercise. Total physical activity was expressed as metabolic equivalent of task (MET)-minutes/week according to established scoring protocols. Based on the responses and the IPAQ guidelines, participants were classified as having a low, moderate, or high level of physical activity. Physical inactivity, used in the tables and as a model covariate, was defined as the low-activity category (<600 MET-min/week).

### 2.2. Clinical, Anthropometric, Biochemical, and Genetic Assessments

Anthropometric data were collected during a single clinic visit according to standard protocols. Height was measured without shoes using a calibrated stadiometer, and weight was recorded using a digital scale with participants wearing light clothing. Body composition was assessed by bioelectrical impedance analysis (BIA; InBody 220, Biospace, Seoul, Republic of Korea), capturing total fat mass, body fat percentage (BF%), fat-free mass, muscle mass, visceral adipose tissue (VAT), and subcutaneous adipose tissue (SAT). Waist and hip circumferences were measured to calculate waist–hip ratio (WHR). All measurements were performed by trained staff, and equipment was routinely calibrated for accuracy.

After an overnight fast of 8–12 h, venous blood samples were collected for measurement of fasting plasma glucose (FPG), insulin, lipid profile parameters (total cholesterol, high-density lipoprotein [HDL] cholesterol, low-density lipoprotein [LDL] cholesterol, and triglycerides), and glycated haemoglobin (HbA1c). A standard 2-h oral glucose tolerance test (OGTT) was also performed, with 75 g of glucose and blood sampling for glucose and insulin at baseline (0 min) and at 30, 60, and 120 min after the glucose load. Glucose and insulin area under the curve (AUC) were calculated using the trapezoidal method based on these four time points (0, 30, 60, and 120 min), or on the three available time points when one sample was missing (13 glucose and 6 insulin curves), and were rescaled to a 0–1 range by min–max scaling across the sample (glucose AUC 5542–46,875 mg/dL·min before scaling). The homeostatic model assessment indices of insulin resistance (HOMA-IR) and β-cell function (HOMA-B) were calculated from fasting glucose and insulin [31].

Genomic DNA was isolated from peripheral blood leukocytes using the classical salting-out method. Target single nucleotide polymorphisms (SNPs) were genotyped using TaqMan SNP technology with a ready-to-use human assay library (Applied Biosystems, Foster City, CA, USA) on the OpenArray high-throughput genotyping platform (Life Technologies, Carlsbad, CA, USA). Candidate variants in BDNF (rs10501087) and TCF7L2 (rs4506565, rs7901695, rs7903146) genes were genotyped as part of the parent project; these genes have established roles in central energy homeostasis (BDNF) and pancreatic β-cell function (TCF7L2). Each SNP was analysed in duplicate with negative controls according to the manufacturer’s instructions. All variants were in Hardy–Weinberg equilibrium (exact test *p* = 0.267 for BDNF rs10501087 and 0.724–0.738 for the TCF7L2 variants), with minor-allele frequencies of 0.201 and 0.235–0.256, respectively. Because rs4506565, rs7901695, and rs7903146 are in strong linkage disequilibrium (pairwise r^2^ 0.77–0.97 in this sample), they were treated as markers of a single TCF7L2 locus. Laboratory personnel were blinded to the clinical and dietary data to minimise bias.

### 2.3. Statistical Analysis

Demographic and clinical characteristics were summarised as means and standard deviations (SDs) for continuous variables and frequencies (percentages) for categorical variables. Descriptive comparisons across coffee-consumption categories (never, weekly, daily) and snacking-frequency categories (rare, frequent) were performed using Kruskal–Wallis tests for three-group comparisons and Mann–Whitney U tests for two-group comparisons for continuous variables, and χ^2^ tests or Fisher’s exact tests, as appropriate, for categorical variables.

Associations of behavioural exposures, namely coffee consumption and snacking frequency, with metabolic outcomes were evaluated using multivariable regression models. Linear regression (ordinary least squares) was used for continuous outcomes (e.g., HDL cholesterol, HbA1c, glucose AUC, and WHR). Associations with prevalent T2DM were examined using logistic regression, with odds ratios (ORs) and 95% confidence intervals (CIs) reported. For consistency, all multivariable models in the primary and sensitivity analyses were, unless stated otherwise, adjusted for the same pre-defined covariate set: age, sex, daily energy intake (kcal), and physical inactivity. To assess potential reverse causation, sensitivity analyses were performed by repeating these adjusted models after excluding participants with prevalent T2DM. For glycaemic outcomes, which were not available for participants with diagnosed T2DM, a further sensitivity analysis excluded participants who met diagnostic thresholds for diabetes at the study visit (fasting glucose ≥ 126 mg/dL, 2-h glucose ≥ 200 mg/dL, or HbA1c ≥ 6.5%). Another sensitivity analysis additionally adjusted for body mass index (BMI), and a further one restricted the AUC outcomes to complete four-point curves. Because energy intake was available for part of the behavioural sample, all models were also fitted without energy intake, which allowed all participants with questionnaire and outcome data to be included (Table S1). For comparison across glycaemic measures, adjusted differences between daily and never coffee consumers were also expressed in standard-deviation units (regression coefficient divided by the pooled within-group standard deviation). Because coffee consumption and snacking may co-occur, their association was tested with a χ^2^ test; models including both exposures simultaneously were fitted, and coffee × snacking interactions were tested. The study had no single pre-specified primary outcome; all analyses were therefore considered exploratory, and Benjamini–Hochberg false discovery rate (FDR)-adjusted q-values are reported alongside nominal *p*-values. Each model used all participants with complete data for its variables (complete-case analysis); no imputation was performed.

Genotype–phenotype associations were assessed by comparing T2DM prevalence across genotypes using χ^2^ tests and by estimating ORs for T2DM using logistic regression: genotype-specific ORs for BDNF rs10501087, with the reference genotype defined a priori, and ORs per minor allele under an additive model for the TCF7L2 variants. The genotype–T2DM ORs were adjusted for age and sex only, because germline genotype cannot be confounded by behavioural covariates. LDL cholesterol was compared across genotypes without adjustment, using Kruskal–Wallis tests. Gene × behaviour interactions were assessed by adding product terms to the fully adjusted behavioural models under an additive genetic model; interaction coefficients with 95% CIs and *p*-values are reported, with FDR correction within this family of tests. Separate multivariable logistic regression models were used to identify predictors of frequent snacking and of never consuming coffee, including age, sex, and genotype variants. Analyses were performed in R software (version 4.6.1; R Foundation for Statistical Computing, Vienna, Austria). All tests were two-tailed, and *p* < 0.05 was considered statistically significant.

## 3. Results

This cross-sectional investigation included an analytic cohort of 1227 participants; coffee and snacking questionnaires were available for 905 participants (729 of them in the analytic cohort), genotype data for 948–993, and energy intake from 3-day dietary records for 464 (Figure S1). Demographic, anthropometric, and metabolic characteristics, stratified by coffee consumption and snacking frequency, are presented in Table 1 and Table 2. Genotype distributions for BDNF and TCF7L2 variants are shown in Table 3, and full multivariable model outputs are provided in Table S1. Regression modelling further indicated demographic patterning of these behaviours: older age and female sex were associated with higher odds of both never consuming coffee (both *p* < 0.001) and frequent snacking (*p* = 0.004 and *p* = 0.005, respectively; Figures S2 and S3).

### 3.1. Glycaemic Excursions and T2DM Prevalence

**Figure 1:**
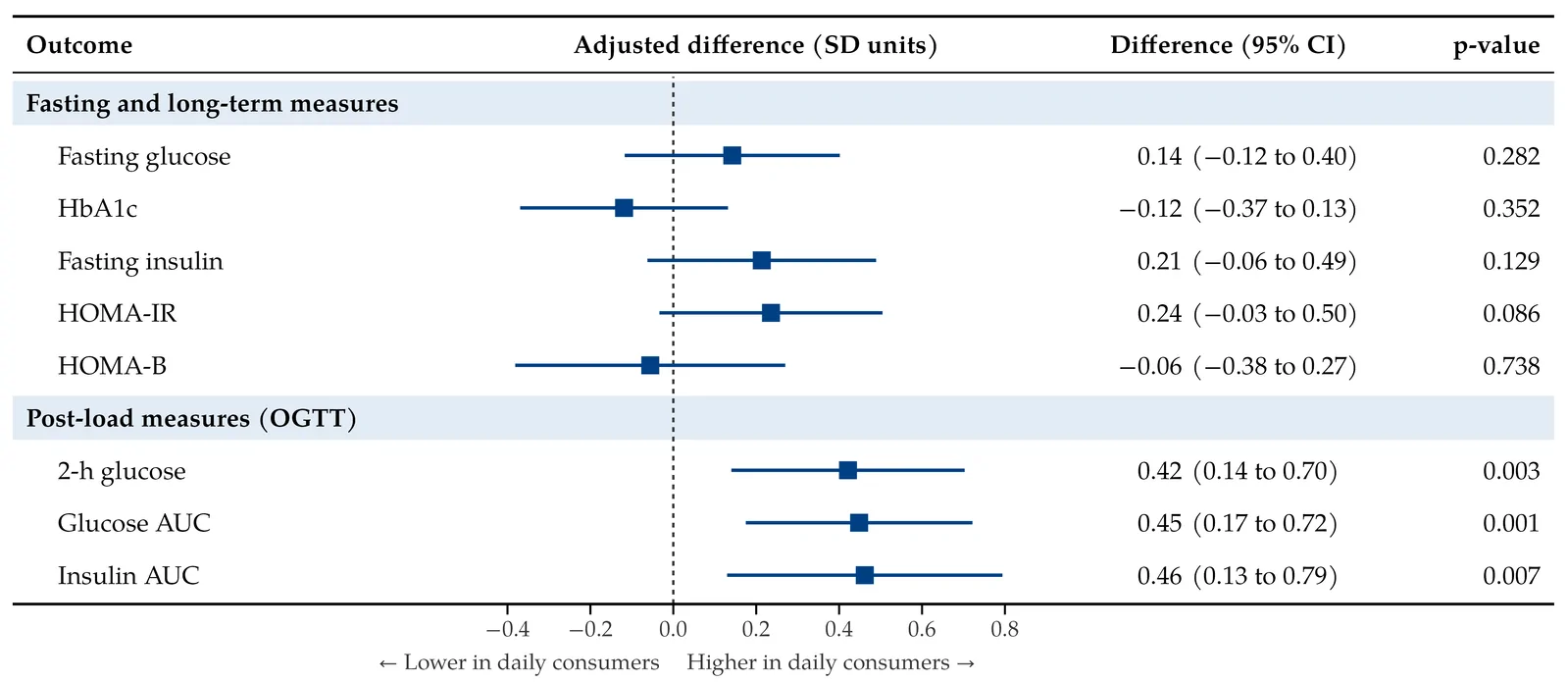
Adjusted differences between daily and never coffee consumers in fasting and long-term glycaemic measures and in post-load measures from the 75 g oral glucose tolerance test (OGTT), expressed in standard-deviation units (regression coefficient divided by the pooled within-group standard deviation), with 95% confidence intervals and *p*-values, from models adjusted for age, sex, daily energy intake, and physical inactivity (Table S1). AUC, area under the curve; CI, confidence interval; HbA1c, glycated haemoglobin; HOMA-B and HOMA-IR, homeostatic model assessment of β-cell function and of insulin resistance; SD, standard deviation.

In unadjusted comparisons, T2DM prevalence was highest among daily coffee consumers (22.5%), compared with never (10.6%) and weekly (8.1%) consumers (*p* = 0.003). In multivariable models adjusted for age, sex, daily energy intake, and physical inactivity, daily coffee consumption (vs. never) was associated with a higher glucose AUC (β = +0.045, 95% CI 0.018–0.073; *p* = 0.001; FDR q = 0.094) and with non-significantly higher odds of prevalent T2DM (OR = 2.76, 95% CI 0.96–7.48; *p* = 0.051); in the larger sample available without adjustment for energy intake (83 rather than 33 participants with T2DM), daily coffee consumption was associated with higher odds of T2DM (OR = 3.30, 95% CI 1.61–6.61; *p* < 0.001; FDR q = 0.058). Daily coffee consumption was also associated with a higher insulin AUC (β = +0.049, 95% CI 0.014–0.084; *p* = 0.007) and 2-h glucose (β = +15.0 mg/dL, 95% CI 5.0–25.1; *p* = 0.003), whereas fasting glucose did not differ (β = +1.9 mg/dL, 95% CI −1.6 to 5.4; *p* = 0.28); in unadjusted means, the glucose difference arose mainly at 30 and 60 min (154 vs. 147 and 139 vs. 131 mg/dL in daily vs. never consumers). The glucose AUC association was similar after additional adjustment for BMI (β = +0.039, 95% CI 0.013–0.065; *p* = 0.003), after mutual adjustment for snacking frequency (β = +0.044, 95% CI 0.017–0.072; *p* = 0.002), without adjustment for energy intake (β = +0.038, 95% CI 0.013–0.063; *p* = 0.003; *n* = 626), and with both AUCs restricted to complete four-point curves (glucose AUC: β = +0.043, 95% CI 0.015–0.071; *p* = 0.002; insulin AUC: β = +0.045, 95% CI 0.010–0.081; *p* = 0.013). Because participants with diagnosed T2DM had no OGTT or HbA1c measurements, the glycaemic models already excluded them; in a sensitivity analysis that additionally excluded 14 participants who met diagnostic thresholds for diabetes at the study visit, the association of daily coffee consumption with glucose AUC was attenuated but persisted (β = +0.035, 95% CI 0.011–0.060; *p* = 0.005). Expressed in standard-deviation units, daily coffee consumption was associated with differences of 0.42–0.46 SD in post-load measures, compared with −0.12 to 0.24 SD in fasting glucose, HbA1c, fasting insulin, and HOMA indices (Figure 1). Coffee consumption frequency was not significantly associated with HbA1c in any model.

For snacking frequency, unadjusted comparisons showed that frequent snacking was associated with higher fasting glucose (94.4 vs. 92.9 mg/dL; *p* = 0.041), HbA1c (5.44% vs. 5.32%; *p* = 0.023), and glucose AUC (0.23 vs. 0.21; *p* = 0.012). After multivariable adjustment, these associations were attenuated and not statistically significant, including for glucose AUC (β = +0.016, 95% CI −0.002 to 0.035; *p* = 0.087; Table S1).

### 3.2. Adiposity and Energy Balance

In descriptive comparisons, frequent snacking was associated with a less favourable adiposity profile. Frequent snackers were older, more often female, and had a higher BMI (28.61 vs. 27.76 kg/m^2^; *p* = 0.039), a greater body fat percentage (32.43% vs. 29.52%; *p* < 0.001), and a higher daily energy intake (1848 vs. 1682 kcal; *p* = 0.017), whereas physical inactivity did not differ significantly (6.1% vs. 2.6%; *p* = 0.062). After multivariable adjustment, frequent snacking remained associated with higher BMI (β = +1.28 kg/m^2^, 95% CI 0.03–2.53; *p* = 0.044), fat mass (β = +2.62 kg, 95% CI 0.01–5.23; *p* = 0.049), and body fat percentage (β = +1.77 percentage points, 95% CI 0.13–3.40; *p* = 0.034; all FDR q = 0.26; Table S1); in the sensitivity model without energy intake, it was associated with a higher WHR (β = +0.012, 95% CI 0.0003–0.024; *p* = 0.044). In the no-T2DM subset, the associations of frequent snacking with BMI (β = +1.18 kg/m^2^, 95% CI −0.13 to 2.49; *p* = 0.078) and body fat percentage (β = +1.68 percentage points, 95% CI −0.05 to 3.41; *p* = 0.056) were of similar magnitude but not statistically significant. For coffee consumption, WHR (*p* = 0.024) and body fat percentage (*p* = 0.026) differed across frequency categories in unadjusted analyses, whereas body weight, BMI, fat mass, and visceral fat did not. Coffee consumption and snacking frequency were not associated with each other: frequent snacking was reported by 68.6%, 65.9%, and 68.5% of never, weekly, and daily coffee consumers (χ^2^ *p* = 0.79; Cramér’s V = 0.02). Including both exposures in the same models did not materially change either set of estimates, and no coffee × snacking interaction remained significant after FDR correction (nominal *p* < 0.05 for glucose AUC, HDL cholesterol, fasting glucose, and 2-h glucose; all q > 0.14).

### 3.3. Lipid Phenotypes

In multivariable models, weekly coffee consumption was associated with higher HDL cholesterol (β = +4.75 mg/dL, 95% CI 1.49–8.02; *p* = 0.004; FDR q = 0.10) relative to never consumption, whereas daily consumption was not (*p* = 0.294). This association between weekly coffee consumption and HDL cholesterol was similar in the no-T2DM subset (β = +5.12 mg/dL, 95% CI 1.78–8.47; *p* = 0.003) and directionally consistent in the sensitivity model without energy intake (β = +2.63 mg/dL, 95% CI −0.16 to 5.43; *p* = 0.065). Snacking frequency was not associated with lipid parameters in descriptive or adjusted analyses (all *p* > 0.05; Table S1), apart from an inverse association with HDL cholesterol in the model including both exposures (β = −3.01 mg/dL, 95% CI −5.74 to −0.27; *p* = 0.031).

### 3.4. Nutrigenetic Susceptibility and Gene–Behaviour Interactions

In genotype-stratified analyses, T2DM prevalence differed across BDNF rs10501087 genotypes (*p* = 0.018). Using TT as the reference, the CT genotype was nominally associated with lower odds of T2DM (age- and sex-adjusted OR = 0.42, 95% CI 0.21–0.78; *p* = 0.009). None of the TCF7L2 variants (rs4506565, rs7901695, rs7903146) was significantly associated with T2DM (age- and sex-adjusted OR per minor allele 1.11–1.19; all *p* ≥ 0.36). In unadjusted comparisons, LDL cholesterol differed across TCF7L2 rs4506565 and rs7901695 genotypes (*p* = 0.017 and *p* = 0.022), with the lowest values in minor-allele homozygotes (Table 3).

**Figure 2:**
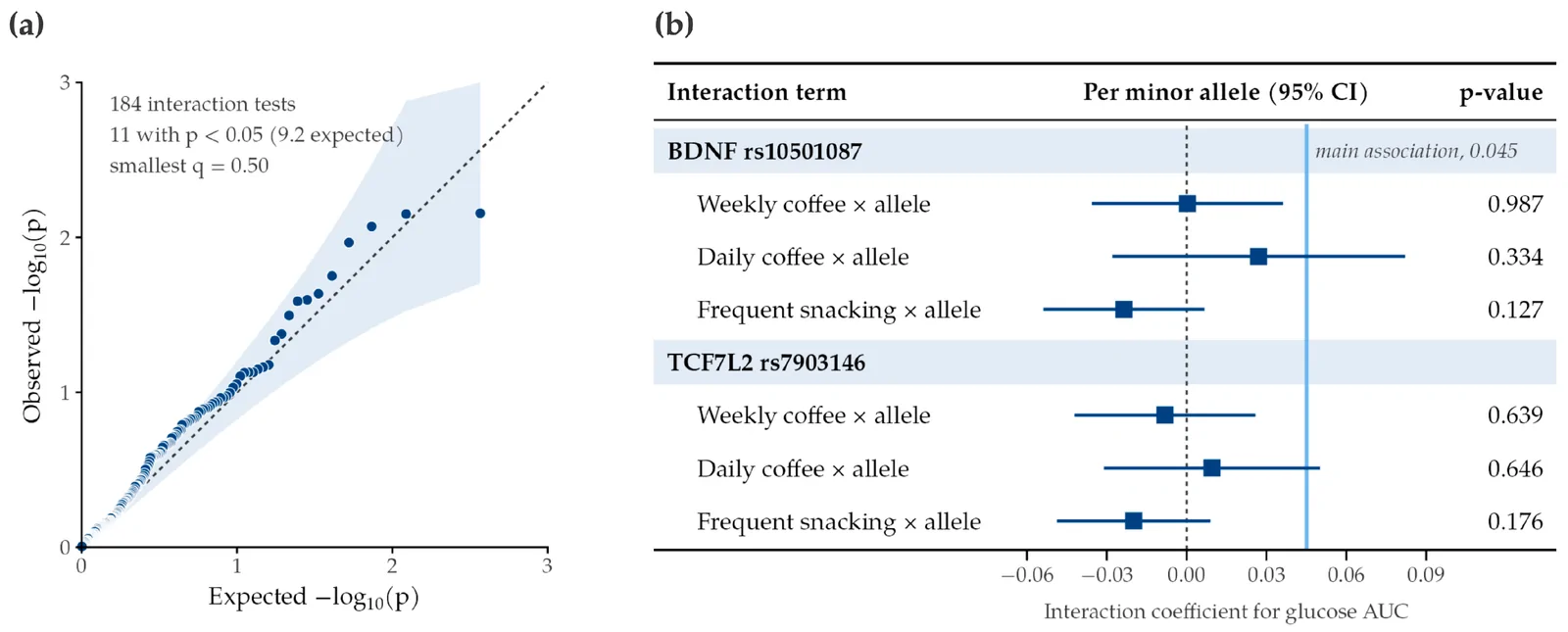
Tests of gene × behaviour interaction: (**a**) quantile–quantile plot of the 184 interaction *p*-values (coffee consumption and snacking frequency × BDNF rs10501087 and TCF7L2 rs4506565, rs7901695, and rs7903146, across 23 outcomes) against their expected distribution in the absence of interaction, with the 95% confidence region shaded; 11 tests had *p* < 0.05 (9.2 expected by chance) and none had q < 0.05, and tests for the three TCF7L2 variants are correlated owing to strong linkage disequilibrium; (**b**) interaction coefficients with 95% confidence intervals and *p*-values for glucose AUC per additional minor allele of BDNF rs10501087 and TCF7L2 rs7903146 (additive genetic model, adjusted for age, sex, daily energy intake, and physical inactivity), with the main association of daily coffee consumption with glucose AUC (β = 0.045) marked for scale; estimates for rs4506565 and rs7901695 were similar (Table S2). AUC, area under the curve; CI, confidence interval.

The associations of coffee consumption and snacking frequency did not differ detectably by BDNF or TCF7L2 genotype: of 184 gene × behaviour interaction tests in the multivariable models, 11 had nominal *p* < 0.05, close to the 9 expected by chance, and none had FDR q < 0.05 (Figure 2a; Table S2). For glucose AUC, the outcome with the strongest behavioural association, none of the interaction coefficients for BDNF or TCF7L2 differed significantly from zero (Figure 2b).

## 4. Discussion

This cross-sectional study addressed whether cardiometabolic traits in Polish adults reflect diet, genes, or both. Within the limits of an exploratory design, the two dietary behaviours studied were more consistently associated with these traits than the BDNF and TCF7L2 variants. The principal finding was a post-load glycaemic pattern among daily coffee consumers. Compared with never consumers, they had higher glucose and insulin responses to the oral glucose load, whereas fasting glucose and HbA1c did not differ after adjustment (Figure 1), and this pattern was consistent after additional adjustment for BMI and for snacking frequency, after excluding participants who met diagnostic thresholds for diabetes at the visit, and without adjustment for energy intake. Because fasting glucose and HbA1c, the glycaemic measures available in many epidemiological studies, did not capture it, the finding illustrates the added value of OGTT-based phenotyping in studies of dietary behaviour. Coffee consumption and snacking frequency were statistically independent in this cohort, so neither association is explained by the other behaviour. Finally, the behavioural associations did not differ detectably by BDNF or TCF7L2 genotype (Figure 2), although modest interactions cannot be excluded.

In descriptive comparisons, snacking frequency showed the most consistent differences. Participants who reported frequent rather than rare snacking had a higher BMI (28.61 vs. 27.76 kg/m^2^), greater fat mass (27.76 vs. 25.20 kg), and a higher body fat percentage (32.43% vs. 29.52%), together with less favourable glycaemic measures, including fasting glucose (94.4 vs. 92.9 mg/dL), HbA1c (5.44% vs. 5.32%), and glucose AUC (0.23 vs. 0.21; all *p* < 0.05). They also consumed more daily energy (approximately 1848 vs 1682 kcal) and derived a lower proportion of energy from protein (19% vs. 20%); physical inactivity did not differ significantly (6.1% vs. 2.6%). In multivariable models adjusted for age, sex, daily energy intake, and physical inactivity, frequent snacking remained nominally associated with higher BMI, fat mass, and body fat percentage (*p* = 0.034–0.049), whereas its glycaemic associations were attenuated. In contrast, associations with coffee consumption varied according to both outcome and frequency of consumption. Weekly coffee consumption was associated with higher HDL cholesterol in multivariable models (β = +4.75 mg/dL, 95% CI 1.49–8.02; *p* = 0.004) relative to never consumption, whereas daily coffee consumption was associated with higher glucose AUC (β = +0.045, 95% CI 0.018–0.073; *p* = 0.001) and non-significantly higher odds of T2DM (OR = 2.76, 95% CI 0.96–7.48; *p* = 0.051 vs. never consumers), but not with HbA1c. Daily coffee consumers also had a non-significantly higher body weight (89.0 vs. 81.9 kg) and BMI (29.53 vs. 28.36 kg/m^2^) and more than double the prevalence of T2DM (22.5% vs. 10.6%, compared with 8.1% among weekly consumers). At the genetic level, the BDNF rs10501087 CT genotype was nominally associated with lower odds of T2DM than the TT genotype, with T2DM prevalence of 5.4% in CT, 12.0% in TT, and 8.6% in CC carriers, although the CC group was too small for a reliable estimate; because this pattern is not monotonic with allele dose, the CT-specific association should be interpreted with caution. The TCF7L2 variants were not significantly associated with T2DM, and no gene × behaviour interaction remained significant after FDR correction.

These findings should be interpreted alongside, rather than in contradiction to, evidence from large prospective studies and meta-analyses of coffee consumption. Pooled prospective analyses have consistently reported that higher habitual coffee consumption is associated with lower incident T2DM risk, with relative risks of approximately 0.70 when the highest and lowest intake categories are compared, together with broadly favourable dose–response relationships [25,32]. These reports also indicate similar inverse associations for caffeinated and decaffeinated coffee, supporting a potential contribution of non-caffeine bioactive compounds [32,33,34]. In this context, the higher glucose AUC observed in our study among daily coffee consumers (0.24 vs. 0.22 in never consumers; adjusted β = +0.045), together with the non-significantly higher odds of prevalent T2DM and the absence of an association with HbA1c, suggests that the metabolic correlates of coffee may be both endpoint-specific and context-dependent. Observational studies have reported findings in the same direction, with higher fasting glucose among coffee consumers [26] and numerically higher coffee intake among patients with T2DM than among controls in a case–control study [35].

Reviews and observational studies indicate that chlorogenic acids and other polyphenols may primarily influence glycaemic regulation, whereas diterpenes retained in unfiltered coffee may more strongly affect lipid fractions [33,36]. HbA1c reflects average glycaemia over approximately 8–12 weeks and is less sensitive to short-term behavioural perturbations than post-load measures. A systematic review of Mendelian randomisation studies found insufficient evidence for an association of genetically predicted coffee consumption with HbA1c but reported associations with higher fasting glucose and insulin resistance and rated the evidence for a higher T2DM risk as probable [37]. The finding of higher glucose AUC but no association with HbA1c among daily coffee consumers in our cohort is compatible with the lower sensitivity of HbA1c, and the direction of the genetic estimates agrees with our post-load findings, although we observed no difference in fasting glucose.

Differences between our cross-sectional estimates and prospective findings on T2DM incidence may additionally reflect exposure misclassification and residual confounding. Previous work has highlighted the misclassification introduced when coffee intake is measured using broad frequency categories, because “one cup” is not standardised, bioactive content varies according to bean type, roast, and brewing method, and information on preparation and additives is often unavailable [27,33,38]. Omission of information on sugar, sweeteners, and creamers further obscures the true exposure because such additives may attenuate or reverse any potential metabolic benefit and introduce additional variability [39]. Collectively, these limitations may dilute associations and contribute to apparent heterogeneity across endpoints [1,38]. In addition, coffee intake clusters with other behaviours, including smoking, energy-dense dietary patterns, and the use of sweetened or “3-in-1” coffee beverages; incomplete adjustment for these correlated behaviours can materially influence estimated coffee–cardiometabolic associations [26,28,37]. In our cohort, daily coffee consumers were more likely to have prevalent T2DM at the time of assessment, suggesting that coffee frequency categories partly capture broader lifestyle and clinical profiles rather than a single isolated exposure. Reverse causation also warrants consideration. Individuals with diagnosed or symptomatic dysglycaemia may modify their diet, including beverage and sugar intake, before or after diagnosis, which may generate paradoxical cross-sectional associations [29]. Participants with diagnosed T2DM had no OGTT or HbA1c measurements, so all glycaemic analyses were confined to participants without diagnosed diabetes, whose coffee habits cannot reflect a change after diagnosis. In a sensitivity analysis that additionally excluded 14 participants who met diagnostic thresholds for diabetes at the study visit (with the same covariates), daily coffee consumption remained associated with a higher glucose AUC (β = +0.035, 95% CI 0.011–0.060; *p* = 0.005) and was not significantly associated with HbA1c. After excluding participants with prevalent T2DM, weekly coffee consumption remained associated with higher HDL cholesterol. These findings suggest that reverse causation, through diagnosis- or symptom-related behavioural change, may contribute to the high prevalence of T2DM among daily coffee consumers, supporting the interpretation of daily coffee consumption, at least in part, as a marker of underlying health status and behavioural adaptation. However, reverse causation does not fully explain their post-load glycaemic profile.

In contrast to the heterogeneous pattern observed for coffee, snacking frequency showed a more consistently adverse cardiometabolic profile in descriptive analyses. Frequent snackers had higher BMI, fat mass, body fat percentage, fasting glucose, HbA1c, glucose AUC, and daily energy intake, and a lower proportion of energy from protein, than those who snacked rarely. Although the absolute differences were modest (approximately 0.9 kg/m^2^ for BMI, 1.5 mg/dL for fasting glucose, and 0.12 percentage points for HbA1c), they were directionally consistent across multiple adverse cardiometabolic parameters. After multivariable adjustment, the adiposity associations remained nominally significant, whereas the glycaemic associations were attenuated and no longer significant; none of the snacking associations survived FDR correction. The descriptive pattern is consistent with evidence that higher consumption of ultra-processed foods, including savoury snacks and sweetened beverages, is associated with a higher risk of T2DM [40,41]. Observational studies [42,43,44] and a review [45] have also linked night eating and later energy intake with less favourable glucose regulation, including higher post-load and postprandial glycaemia, and with T2DM, consistent with circadian patterns in insulin sensitivity and β-cell responsiveness. Although our snacking variable did not distinguish snack timing, the less favourable adiposity and glycaemic profile observed among frequent snackers is consistent with broader evidence suggesting that snacking, particularly when accompanied by higher energy intake and a sedentary lifestyle, may function as a practical marker of cardiometabolic risk.

Importantly, the literature distinguishes snacking frequency from snack quality and timing. Experimental and acute-feeding studies indicate that protein- and fibre-rich foods, such as nuts, can attenuate postprandial glycaemic excursions [45], whereas time-restricted eating and earlier distribution of energy intake have been associated with modest reductions in body weight, BMI, and waist circumference [46]. Our questionnaire-derived snacking variable captured frequency alone and therefore likely encompassed heterogeneous behaviours, ranging from low-quality evening snacking to more structured and nutritionally balanced between-meal eating. This heterogeneity may help explain the relatively modest differences in lipid measures and insulin AUC despite significant differences in adiposity and glucose metrics. Our adjusted models further indicate that the glycaemic associations of snacking were attenuated after accounting for age, sex, energy intake, and physical inactivity, suggesting confounding by age and sex and possibly mediation through energy intake. Nonetheless, the finding that a simple dichotomy of frequent versus rare snacking identified a less favourable metabolic profile in descriptive analyses is consistent with the use of snacking frequency as a pragmatic behavioural marker of cardiometabolic risk and with studies linking snack intake and eating frequency with metabolic syndrome [47,48].

The genetic analyses provide preliminary data on background susceptibility. In our data, the BDNF rs10501087 CT genotype was nominally associated with lower odds of T2DM than the TT genotype, reflected in lower T2DM prevalence (5.4% vs. 12.0%, with 8.6% in CC carriers). This observation is compatible with evidence that BDNF is involved in both brain function and regulation of energy balance [18], that peripheral BDNF concentrations in T2DM and obesity have been reported as both higher and lower than in controls [21,24,49], and that BDNF haploinsufficiency or deficiency promotes hyperphagia and obesity in experimental models [11,19]. Because rs10501087 has been studied mainly in relation to BMI and obesity [12,50], the lower odds of T2DM in CT carriers represent a hypothesis-generating observation that merits replication and mechanistic investigation. For TCF7L2, we did not observe clear associations with T2DM or glycaemic markers in this cross-sectional analysis, although larger studies have extensively linked TCF7L2 risk alleles to impaired insulin secretion, OGTT-derived glycaemic curves, and incident T2DM [13,15,51]. This difference is plausible because HbA1c and fasting cross-sectional measures are susceptible to metabolic compensation, treatment effects, and behavioural modification, whereas TCF7L2 effects are most consistently detected for incident T2DM and indices of β-cell function [13,51]. The modest number of T2DM cases in our cohort, together with the inclusion of several SNPs and covariates, further limited statistical power to detect small genetic signals, particularly for less frequent genotypes, such as BDNF rs10501087 CC.

Gene–diet interaction analyses are particularly demanding methodologically. Interaction tests generally require substantially larger sample sizes than analyses of main effects [52]. Moreover, error in self-reported dietary exposures reduces the power to detect gene × diet interactions [52]. Existing nutrigenetic studies have reported interactions of BDNF variants with energy and protein intake [12,53] and of TCF7L2 variants with dietary fibre and macronutrient intake [54,55], suggesting that the metabolic correlates of these variants may depend on dietary context. For coffee, an interaction with human leukocyte antigen genotype has been described for latent autoimmune diabetes in adults [4], whereas in the EPIC-InterAct study, a nominal interaction between TCF7L2 rs12255372 and coffee intake (*p* = 0.048) did not survive correction for multiple testing [56]. In the Maastricht Study, coffee consumption did not interact with a polygenic risk score for T2DM, whereas higher consumption was associated with lower odds of prediabetes and T2DM, a main effect that contrasts with the association between daily coffee consumption and glucose AUC observed here [5]. In our data, no interaction of coffee or snacking with BDNF or TCF7L2 genotype survived FDR correction (11 of 184 tests had nominal *p* < 0.05, close to the 9 expected by chance). This pattern is consistent with methodological work showing that small gene × diet effects are difficult to detect under substantial dietary measurement error and limited sample size [52]. It also mirrors randomised evidence that genotype did not modify weight loss on healthy low-fat versus low-carbohydrate diets [57] and that adding genotype to personalised nutrition advice did not enhance dietary change [58], as well as the limited replicability of candidate gene × environment findings [59]. Collectively, these findings indicate that BDNF and TCF7L2 genotypes are not yet a suitable basis for dietary guidance on coffee or snacking.

Taken together, our data suggest that the metabolic associations of coffee consumption and snacking frequency are endpoint-specific rather than uniformly “protective” or “harmful”. In this cohort, snacking frequency, rather than coffee frequency, showed the clearest adverse metabolic profile in descriptive analyses, encompassing higher adiposity, less favourable glycaemic indices, and higher energy intake. Coffee consumption and snacking frequency also differed according to age and sex, and the daily coffee category included a higher proportion of participants with prevalent T2DM, suggesting that these frequency categories reflect broader lifestyle patterns and disease-related behavioural modification. The adjusted and sensitivity analyses refine this interpretation. Snacking frequency appeared to capture a higher-risk lifestyle cluster in descriptive comparisons; its associations with adiposity remained nominally significant after adjustment (but not in the no-T2DM subset), and none survived FDR correction. In contrast, the association of daily coffee consumption with glucose AUC persisted after excluding participants who met diagnostic thresholds for diabetes, and the association of weekly coffee consumption with HDL cholesterol was similar after exclusion of prevalent T2DM. This interpretation is consistent with evidence that coffee and other beverage habits co-occur with smoking [28], overall diet quality, and use of sweetened coffee products [26,39], and that incomplete control of such behavioural clustering can mask, exaggerate, or reverse coffee–cardiometabolic associations [1,37]. Within this framework, BDNF and TCF7L2 are best regarded as background susceptibility loci within which everyday behaviours operate, rather than as loci that currently justify genotype-tailored advice on coffee consumption or snacking frequency, although the study was not powered to exclude modest interactions.

This study has several strengths and limitations. Strengths include the simultaneous assessment of coffee consumption, snacking frequency, detailed anthropometric indices, OGTT-derived glucose and insulin AUC, HbA1c, and lipid profile, together with genotyping of BDNF rs10501087 and multiple TCF7L2 variants (analytic cohort, *n* = 1227; behavioural sample, *n* = 905; genotyped, *n* = 948–993), with energy intake from 3-day dietary records in a subsample (*n* = 464). This dietary subsample enabled adjustment for energy intake and evaluation of dietary composition, and the multivariable models incorporated both behavioural and genetic factors. The four-point OGTT with paired insulin measurements captured the post-load glucose and insulin response, which fasting glucose and HbA1c do not reflect, and the principal glycaemic association was consistent after additional adjustment for BMI and after mutual adjustment for coffee and snacking. Gene × behaviour interactions were tested formally for every exposure–outcome combination, genotype data were checked for Hardy–Weinberg equilibrium, and FDR-adjusted q-values are reported for the primary behavioural models and all interaction tests, so these findings can be read against the full set of tests performed. However, the cross-sectional design precludes causal inference, and reverse causation may explain some observed patterns, particularly the higher prevalence of T2DM among daily coffee consumers [29]. Accordingly, we performed sensitivity analyses excluding prevalent T2DM and, for glycaemic outcomes, participants who met diagnostic thresholds for diabetes at the study visit, using the same adjustment set as in the primary models; however, residual confounding and exposure misclassification remain plausible, and the reduced sample size likely limited precision for some estimates. Energy intake was available for 464 of the 905 participants with behavioural data, and the principal glycaemic association was similar with and without adjustment for energy intake (Table S1). Coffee and snacking behaviours were assessed using self-reported questionnaires with broad frequency categories and without information on cup size, brewing method, additives, snack composition, or snack timing, all of which have been identified as sources of misclassification in dietary studies [27,52,60]. Residual confounding is also likely because we could not fully account for behaviours that cluster with coffee and snacking, including smoking, sleep, socioeconomic factors, adiposity trajectories, broader diet quality, and details of coffee preparation and additives, which have been highlighted as influential covariates in models of coffee, snacking, and metabolic outcomes [28,37,48]. The single-country setting may additionally limit generalisability to other populations. Finally, the number of T2DM cases and the distribution of some genotypes restricted power for the genetic and gene × behaviour analyses, and methodological work indicates that much larger cohorts with more precise dietary exposure assessment are required to obtain stable interaction estimates [52].

The present findings have several implications. First, the post-load pattern observed among daily coffee consumers suggests that OGTT-based phenotyping may detect diet-related metabolic differences that fasting glucose and HbA1c miss. This is relevant to the design of future nutritional studies and potentially to clinical assessment, because post-load glucose carries prognostic information that fasting glucose alone does not capture [61], and 1-h post-load glucose has been proposed as a more sensitive marker of risk than fasting glucose or HbA1c [62]. Second, the association of frequent snacking with higher adiposity, and descriptively with less favourable glycaemic traits, suggests that snacking behaviour, particularly when interpreted alongside information on diet quality and beverage choices, may serve as a practical target for lifestyle counselling and as a behavioural marker of cardiometabolic risk. Third, the endpoint-specific associations observed for coffee, set against the inverse associations reported in prospective studies of incident T2DM, underscore the value of interpreting coffee consumption within its broader lifestyle and clinical context rather than as a uniformly health-promoting or risk-promoting behaviour. The associations of daily coffee consumption with glucose AUC and of weekly coffee consumption with HDL cholesterol are best interpreted as endpoint-specific signals that warrant prospective confirmation. Fourth, the absence of detectable gene × behaviour interactions suggests that, at present, BDNF and TCF7L2 genotypes add little to behavioural and metabolic phenotyping as a basis for dietary guidance, although modest interactions cannot be excluded; the nominal BDNF association identifies a candidate for replication. Future studies should replicate these behavioural and genetic patterns in larger prospective cohorts with repeated and more granular assessments of coffee preparation, additives, snack quality, and timing, while incorporating more comprehensive adjustment for lifestyle and socioeconomic confounders. Gene–diet interactions should then be evaluated in adequately powered designs. Genotype-stratified or enrichment-based intervention studies, particularly those combining refined dietary phenotyping with mechanistic endpoints, such as OGTT-derived curves and β-cell function indices, could clarify whether specific combinations of coffee consumption and snacking behaviours modify T2DM risk beyond their role as markers of broader lifestyle patterns.

## 5. Conclusions

In this cohort of Polish adults, everyday dietary behaviour was more consistently related to cardiometabolic traits than BDNF or TCF7L2 genotype. Frequent snacking appeared to mark a broader lifestyle pattern associated with higher adiposity and may serve as a practical behavioural marker of cardiometabolic risk. In contrast, coffee consumption showed frequency-specific associations: daily consumption was associated with a higher post-load glucose and insulin response that fasting glucose and HbA1c did not capture, and weekly consumption with higher HDL cholesterol.

At the genetic level, the BDNF rs10501087 CT genotype was nominally associated with lower odds of T2DM, identifying a potential direction for future mechanistic investigation. Because the behavioural associations did not differ detectably by BDNF or TCF7L2 genotype, the current findings do not support genotype-specific dietary recommendations regarding coffee consumption or snacking; instead, they highlight OGTT-based phenotyping as a sensitive tool for detecting diet-related metabolic differences. Whether interpreting these habits within the broader context of energy balance, diet quality, and lifestyle improves cardiometabolic risk assessment should be tested in prospective studies.

## Figures and Tables

**Table 1:** Participant characteristics according to coffee consumption frequency (never, weekly, daily).

Variable	Never (*n* = 612)	Weekly (*n* = 182)	Daily (*n* = 111)	*p*-Value
Age (years)	42.58 ± 14.28	35.59 ± 14.11	37.24 ± 14.94	<0.001
Female sex	309/519 (59.5%)	62/135 (45.9%)	34/80 (42.5%)	<0.001
Body weight (kg)	81.86 ± 21.07	82.84 ± 21.51	89.00 ± 26.03	0.083
Height (m)	1.70 ± 0.09	1.72 ± 0.09	1.73 ± 0.09	<0.001
BMI (kg/m^2^)	28.36 ± 6.45	27.75 ± 6.71	29.53 ± 7.71	0.290
Waist–hip ratio (WHR)	0.92 ± 0.08	0.93 ± 0.09	0.95 ± 0.10	0.024
Fat mass (kg)	27.07 ± 13.19	25.54 ± 13.30	29.19 ± 17.70	0.250
Body fat (%)	32.10 ± 9.59	29.70 ± 9.86	31.28 ± 10.11	0.026
Fat-free mass (kg)	54.62 ± 12.80	57.30 ± 13.31	59.55 ± 13.33	0.002
Muscle mass (kg)	25.61 ± 7.29	27.63 ± 7.41	28.71 ± 7.64	0.002
Visceral fat (%)	37.48 ± 12.01	35.64 ± 11.74	37.65 ± 12.83	0.375
Subcutaneous fat (%)	62.54 ± 11.97	64.36 ± 11.74	62.35 ± 12.83	0.376
Fasting glucose (mg/dL)	94.29 ± 14.05	92.82 ± 11.23	94.36 ± 12.45	0.557
Glucose at 120 min (mg/dL)	99.59 ± 37.14	98.91 ± 27.98	103.24 ± 38.04	0.380
HbA1c (%)	5.44 ± 0.60	5.32 ± 0.52	5.26 ± 0.50	0.033
T2DM prevalence	55/518 (10.6%)	11/135 (8.1%)	18/80 (22.5%)	0.003
Glucose AUC	0.22 ± 0.10	0.22 ± 0.09	0.24 ± 0.10	0.235
Insulin AUC	0.13 ± 0.10	0.15 ± 0.13	0.17 ± 0.12	0.018
Total cholesterol (mg/dL)	195.24 ± 41.86	185.44 ± 38.32	188.36 ± 40.45	0.060
LDL cholesterol (mg/dL)	112.28 ± 38.99	102.71 ± 34.57	106.81 ± 35.85	0.043
HDL cholesterol (mg/dL)	60.77 ± 15.30	62.26 ± 15.21	56.74 ± 12.27	0.052
Triglycerides (mg/dL)	110.92 ± 67.81	102.41 ± 53.19	123.28 ± 77.73	0.253
Daily energy intake (kcal)	1808.81 ± 690.70	1817.09 ± 746.40	1693.15 ± 665.48	0.533
Protein intake (% energy)	19 ± 5	19 ± 4	19 ± 4	0.852
Carbohydrate intake (% energy)	47 ± 9	48 ± 8	48 ± 9	0.498
Fat intake (% energy)	31 ± 7	31 ± 8	32 ± 8	0.811
Physical inactivity	23/519 (4.4%)	9/135 (6.7%)	5/80 (6.2%)	0.499

Data are presented as mean ± SD or *n*/*N* (%). Group differences were assessed using Kruskal–Wallis tests for continuous variables and χ^2^ tests (or Fisher’s exact tests where appropriate) for categorical variables. Analyses included participants with available coffee frequency data. Glucose and insulin AUC are shown after min–max scaling to 0–1 (Section 2.2). AUC, area under the curve; BMI, body mass index; HbA1c, glycated haemoglobin; HDL, high-density lipoprotein; LDL, low-density lipoprotein; SD, standard deviation; T2DM, type 2 diabetes mellitus; WHR, waist–hip ratio.

**Table 2:** Participant characteristics according to snacking frequency (rare vs. frequent).

Variable	Rare Snacking (*n* = 292)	Frequent Snacking (*n* = 616)	*p*-Value
Age (years)	38.77 ± 14.98	41.26 ± 14.36	0.009
Female sex	106/232 (45.7%)	300/505 (59.4%)	<0.001
Body weight (kg)	82.36 ± 22.14	82.97 ± 21.69	0.608
Height (m)	1.72 ± 0.09	1.70 ± 0.09	0.006
BMI (kg/m^2^)	27.76 ± 6.64	28.61 ± 6.65	0.039
Waist–hip ratio (WHR)	0.92 ± 0.09	0.93 ± 0.09	0.388
Fat mass (kg)	25.20 ± 13.83	27.76 ± 13.65	0.002
Body fat (%)	29.52 ± 9.69	32.43 ± 9.59	<0.001
Fat-free mass (kg)	56.90 ± 12.97	55.09 ± 13.06	0.099
Muscle mass (kg)	27.34 ± 7.47	26.00 ± 7.38	0.131
Visceral fat (%)	36.60 ± 10.82	37.41 ± 12.56	0.886
Subcutaneous fat (%)	63.40 ± 10.82	62.62 ± 12.52	0.886
Fasting glucose (mg/dL)	92.89 ± 12.18	94.44 ± 13.88	0.041
Glucose at 120 min (mg/dL)	95.75 ± 31.91	101.30 ± 36.84	0.052
HbA1c (%)	5.32 ± 0.50	5.44 ± 0.61	0.023
Glucose AUC	0.21 ± 0.09	0.23 ± 0.10	0.012
Insulin AUC	0.13 ± 0.10	0.14 ± 0.11	0.446
Total cholesterol (mg/dL)	194.25 ± 45.77	192.09 ± 39.93	0.673
LDL cholesterol (mg/dL)	111.92 ± 41.03	109.14 ± 37.23	0.456
HDL cholesterol (mg/dL)	60.59 ± 14.17	60.55 ± 15.43	0.880
Triglycerides (mg/dL)	108.68 ± 63.25	111.86 ± 69.16	0.619
Daily energy intake (kcal)	1681.54 ± 665.31	1847.54 ± 706.62	0.017
Protein intake (% energy)	20 ± 5	19 ± 5	0.045
Carbohydrate intake (% energy)	47 ± 8	48 ± 9	0.476
Fat intake (% energy)	31 ± 8	31 ± 7	0.396
Physical inactivity	6/232 (2.6%)	31/505 (6.1%)	0.062

Data are presented as mean ± SD or *n*/*N* (%). Between-group differences were assessed using Mann–Whitney U tests for continuous variables and χ^2^ tests (or Fisher’s exact tests where appropriate) for categorical variables. Analyses included participants with available snacking frequency data. Glucose and insulin AUC are shown after min–max scaling to 0–1 (Section 2.2). AUC, area under the curve; BMI, body mass index; HbA1c, glycated haemoglobin; HDL, high-density lipoprotein; LDL, low-density lipoprotein; SD, standard deviation; WHR, waist–hip ratio.

**Table 3:** Genotype distributions, type 2 diabetes (T2DM) prevalence, and LDL cholesterol levels according to BDNF rs10501087 and TCF7L2 variants (rs4506565, rs7901695, rs7903146). T2DM prevalence is presented as *n*/*N* (%) among participants with known T2DM status (χ^2^ tests: BDNF *p* = 0.018; TCF7L2 *p* = 0.71–0.85) and LDL as mean ± SD (Kruskal–Wallis tests: BDNF *p* = 0.666; TCF7L2 rs4506565 *p* = 0.017, rs7901695 *p* = 0.022, rs7903146 *p* = 0.065). LDL, low-density lipoprotein; SD, standard deviation; SNP, single nucleotide polymorphism.

SNP	Genotype	*n*	T2DM, *n*/*N* (%)	LDL (mg/dL), Mean ± SD
BDNF rs10501087	CC	44	3/35 (8.6%)	115.2 ± 38.5
BDNF rs10501087	CT	294	13/239 (5.4%)	110.7 ± 39.9
BDNF rs10501087	TT	610	60/498 (12.0%)	112.1 ± 40.7
TCF7L2 rs4506565	AA	546	46/430 (10.7%)	114.9 ± 42.4
TCF7L2 rs4506565	AT	382	38/306 (12.4%)	109.6 ± 38.2
TCF7L2 rs4506565	TT	62	6/49 (12.2%)	98.1 ± 31.1
TCF7L2 rs7901695	CC	61	6/49 (12.2%)	98.1 ± 31.1
TCF7L2 rs7901695	CT	379	37/305 (12.1%)	109.7 ± 37.4
TCF7L2 rs7901695	TT	552	47/433 (10.9%)	114.8 ± 42.9
TCF7L2 rs7903146	CC	583	48/459 (10.5%)	113.7 ± 41.7
TCF7L2 rs7903146	CT	353	35/283 (12.4%)	110.2 ± 38.2
TCF7L2 rs7903146	TT	57	5/48 (10.4%)	100.4 ± 37.5

## Data Availability

The data presented in this study are available on request from the corresponding author due to privacy and ethical restrictions relating to participant confidentiality.

## Supplementary Materials

The following supporting information can be downloaded at: https://www.mdpi.com/article/10.3390/nu18213545/s1. Table S1: Multivariable-adjusted associations of habitual coffee consumption and snacking frequency with anthropometric, glycaemic, and lipid outcomes, with false discovery rate-adjusted q-values. Table S2: Gene × behaviour interaction estimates for BDNF and TCF7L2 variants. Figure S1: Participant flow diagram. Figure S2: Coffee consumption and snacking frequency by sex. Figure S3: Coffee consumption and snacking frequency by age group.

## Abbreviations

The following abbreviations are used in this manuscript:

AUC	area under the curve
BDNF	brain-derived neurotrophic factor
BF%	body fat percentage
BIA	bioelectrical impedance analysis
BMI	body mass index
CI	confidence interval
FDR	false discovery rate
FPG	fasting plasma glucose
HbA1c	glycated haemoglobin
HDL	high-density lipoprotein (cholesterol)
HOMA-B	homeostatic model assessment of β-cell function
HOMA-IR	homeostatic model assessment of insulin resistance
IPAQ-LF	International Physical Activity Questionnaire-Long Form
LDL	low-density lipoprotein (cholesterol)
MET	metabolic equivalent of task
OGTT	oral glucose tolerance test
OR	odds ratio
SAT	subcutaneous adipose tissue
SD	standard deviation
SNP	single nucleotide polymorphism
T2DM	type 2 diabetes mellitus
TCF7L2	transcription factor 7-like 2
VAT	visceral adipose tissue
WHR	waist–hip ratio
