# Supplementary material for: Diet, Genes, or Both? Coffee Consumption, Snacking, and BDNF and TCF7L2 Genotypes in Relation to Cardiometabolic Traits: A Cross-Sectional Study in Polish Adults
Source: Nutrients. 2026 Oct 27;18(21):3545. doi: 10.3390/nu18213545 (PMC13764798; doi:10.3390/nu18213545)
Supplement: Supplementary file 1 [file nutrients-18-03545-s001.zip › nutrients-4459711-supplementary.pdf]

Supplementary Materials

Diet, Genes, or Both? Coffee Consumption, Snacking, and BDNF and TCF7L2 Genotypes in Relation to Cardiometabolic Traits: A Cross-Sectional Study in Polish Adults

Table S1. Multivariable-adjusted associations of habitual coffee consumption and snacking frequency with anthropometric, glycaemic, and lipid outcomes, with false discovery rate-adjusted q-values.

		Primary model: age, sex, daily energy intake, physical inactivity				Sensitivity model: age, sex, physical inactivity (no energy intake)			
**Outcome**	**Contrast**	**β or OR (95% CI)**	**p**	**q**	**n**	**β or OR (95% CI)**	**p**	**q**	**n**
Coffee consumption (reference: never)									
Body weight (kg)	Weekly vs never	−1.49 (−5.97 to 3.00)	0.515	0.661	464	−0.29 (−3.95 to 3.37)	0.877	0.895	730
	Daily vs never	3.35 (−1.97 to 8.66)	0.217	0.498	464	5.02 (0.48 to 9.56)	0.030	0.208	730
BMI (kg/m²)	Weekly vs never	−0.62 (−2.11 to 0.88)	0.416	0.636	464	−0.14 (−1.34 to 1.07)	0.825	0.890	730
	Daily vs never	0.74 (−1.03 to 2.51)	0.412	0.636	464	1.44 (−0.05 to 2.93)	0.059	0.237	730
Fat mass (kg)	Weekly vs never	−0.81 (−3.93 to 2.31)	0.610	0.707	462	0.20 (−2.34 to 2.74)	0.877	0.895	724
	Daily vs never	1.89 (−1.83 to 5.61)	0.319	0.551	462	3.64 (0.44 to 6.85)	0.026	0.204	724
Body fat (%)	Weekly vs never	−0.42 (−2.38 to 1.53)	0.670	0.730	462	0.21 (−1.35 to 1.77)	0.788	0.877	724
	Daily vs never	0.68 (−1.65 to 3.02)	0.565	0.684	462	1.89 (−0.08 to 3.86)	0.060	0.237	724
Fat-free mass (kg)	Weekly vs never	−0.45 (−2.35 to 1.46)	0.645	0.718	462	−0.30 (−1.84 to 1.24)	0.701	0.820	724
	Daily vs never	1.27 (−1.00 to 3.54)	0.272	0.540	462	1.19 (−0.76 to 3.13)	0.231	0.411	724
Muscle mass (kg)	Weekly vs never	−0.44 (−1.38 to 0.50)	0.356	0.586	333	−0.18 (−0.97 to 0.61)	0.653	0.794	486
	Daily vs never	0.79 (−0.29 to 1.87)	0.149	0.428	333	0.69 (−0.29 to 1.66)	0.167	0.391	486
Waist circumference (cm)	Weekly vs never	−0.52 (−4.15 to 3.11)	0.777	0.800	464	0.68 (−2.23 to 3.60)	0.647	0.794	727
	Daily vs never	2.52 (−1.79 to 6.82)	0.251	0.540	464	4.44 (0.81 to 8.08)	0.017	0.190	727
Hip circumference (cm)	Weekly vs never	−1.32 (−4.22 to 1.57)	0.369	0.592	464	−0.10 (−2.40 to 2.20)	0.931	0.931	727
	Daily vs never	1.78 (−1.65 to 5.21)	0.309	0.551	464	1.95 (−0.92 to 4.81)	0.183	0.391	727
Waist–hip ratio	Weekly vs never	0.0065 (−0.0118 to 0.0248)	0.487	0.659	464	0.0066 (−0.0079 to 0.0211)	0.371	0.567	727
	Daily vs never	0.0065 (−0.0152 to 0.0282)	0.555	0.684	464	0.0233 (0.0053 to 0.0413)	0.011	0.184	727
Visceral fat (%)	Weekly vs never	−3.44 (−6.87 to −0.004)	0.0498	0.262	333	−2.09 (−4.87 to 0.69)	0.141	0.359	486
	Daily vs never	0.98 (−2.95 to 4.92)	0.623	0.707	333	−0.26 (−3.69 to 3.18)	0.882	0.895	486
Fasting glucose (mg/dL)	Weekly vs never	0.28 (−2.48 to 3.04)	0.842	0.854	428	−0.24 (−2.71 to 2.24)	0.849	0.895	644
	Daily vs never	1.91 (−1.57 to 5.39)	0.282	0.540	428	1.95 (−1.40 to 5.31)	0.253	0.416	644
2-h glucose (mg/dL)	Weekly vs never	8.11 (0.16 to 16.07)	0.046	0.262	426	5.55 (−1.20 to 12.30)	0.107	0.308	637
	Daily vs never	15.03 (4.99 to 25.06)	0.003	0.101	426	11.57 (2.41 to 20.73)	0.013	0.184	637
Glucose AUC (scaled 0–1)	Weekly vs never	0.0158 (−0.0061 to 0.0377)	0.157	0.433	418	0.0070 (−0.0116 to 0.0256)	0.461	0.650	626
	Daily vs never	0.0451 (0.0176 to 0.0726)	0.001	0.094	418	0.0381 (0.0130 to 0.0633)	0.003	0.070	626
HbA1c (%)	Weekly vs never	−0.02 (−0.13 to 0.10)	0.759	0.794	428	−0.03 (−0.14 to 0.08)	0.561	0.731	643
	Daily vs never	−0.07 (−0.21 to 0.08)	0.352	0.586	428	−0.07 (−0.21 to 0.08)	0.379	0.567	643
Fasting insulin (μIU/mL)	Weekly vs never	2.08 (0.06 to 4.10)	0.043	0.262	428	1.30 (−0.57 to 3.17)	0.173	0.391	645
	Daily vs never	1.97 (−0.58 to 4.52)	0.129	0.405	428	2.94 (0.40 to 5.47)	0.023	0.204	645

Outcome	Contrast	Primary model: age, sex, daily energy intake, physical inactivity				Sensitivity model: age, sex, physical inactivity (no energy intake)			
		β or OR (95% CI)	p	q	n	β or OR (95% CI)	p	q	n
Insulin AUC (scaled 0–1)	Weekly vs never	0.0229 (−0.0052 to 0.0510)	0.110	0.381	415	0.0209 (−0.0011 to 0.0429)	0.063	0.237	622
	Daily vs never	0.0491 (0.0138 to 0.0844)	0.007	0.113	415	0.0476 (0.0178 to 0.0773)	0.002	0.061	622
HOMA-IR	Weekly vs never	0.53 (0.02 to 1.05)	0.042	0.262	428	0.31 (−0.17 to 0.79)	0.208	0.396	644
	Daily vs never	0.57 (−0.08 to 1.22)	0.086	0.315	428	0.74 (0.09 to 1.40)	0.027	0.204	644
HOMA-B	Weekly vs never	14.74 (−25.95 to 55.43)	0.477	0.658	427	5.90 (−25.85 to 37.66)	0.715	0.822	643
	Daily vs never	−8.76 (−60.08 to 42.57)	0.738	0.783	427	9.56 (−33.49 to 52.60)	0.663	0.794	643
Total cholesterol (mg/dL)	Weekly vs never	−3.18 (−12.81 to 6.46)	0.517	0.661	435	−4.99 (−12.83 to 2.84)	0.211	0.396	677
	Daily vs never	−2.81 (−14.07 to 8.46)	0.625	0.707	435	−2.88 (−12.62 to 6.85)	0.561	0.731	677
LDL cholesterol (mg/dL)	Weekly vs never	−6.19 (−15.14 to 2.76)	0.175	0.463	435	−5.76 (−12.98 to 1.46)	0.118	0.325	677
	Daily vs never	−4.02 (−14.48 to 6.44)	0.450	0.636	435	−2.53 (−11.50 to 6.45)	0.580	0.742	677
HDL cholesterol (mg/dL)	Weekly vs never	4.75 (1.49 to 8.02)	0.004	0.101	435	2.63 (−0.16 to 5.43)	0.065	0.237	678
	Daily vs never	−2.04 (−5.86 to 1.78)	0.294	0.548	435	−2.32 (−5.77 to 1.13)	0.187	0.391	678
Triglycerides (mg/dL)	Weekly vs never	−8.70 (−24.40 to 7.00)	0.277	0.540	435	−9.55 (−22.05 to 2.94)	0.134	0.355	679
	Daily vs never	16.27 (−2.08 to 34.63)	0.082	0.315	435	9.02 (−6.46 to 24.50)	0.253	0.416	679
Prevalent T2DM	Weekly vs never	OR 0.78 (0.20 to 2.35)	0.677	0.730	464 (33)	OR 0.88 (0.41 to 1.78)	0.737	0.833	732 (83)
	Daily vs never	OR 2.76 (0.96 to 7.48)	0.051	0.262	464 (33)	OR 3.30 (1.61 to 6.61)	<0.001	0.058	732 (83)
Snacking frequency (reference: rare)									
Body weight (kg)	Frequent vs rare	3.74 (−0.02 to 7.49)	0.051	0.262	465	2.91 (−0.09 to 5.92)	0.057	0.237	733
BMI (kg/m²)	Frequent vs rare	1.28 (0.03 to 2.53)	0.044	0.262	465	0.85 (−0.13 to 1.84)	0.091	0.284	733
Fat mass (kg)	Frequent vs rare	2.62 (0.01 to 5.23)	0.049	0.262	463	1.88 (−0.21 to 3.97)	0.078	0.268	727
Body fat (%)	Frequent vs rare	1.77 (0.13 to 3.40)	0.034	0.262	463	1.21 (−0.08 to 2.49)	0.065	0.237	727
Fat-free mass (kg)	Frequent vs rare	1.24 (−0.36 to 2.83)	0.128	0.405	463	1.20 (−0.07 to 2.46)	0.064	0.237	727
Muscle mass (kg)	Frequent vs rare	0.52 (−0.27 to 1.32)	0.196	0.482	334	0.23 (−0.43 to 0.89)	0.487	0.672	487
Waist circumference (cm)	Frequent vs rare	2.74 (−0.29 to 5.78)	0.076	0.315	465	2.11 (−0.29 to 4.51)	0.085	0.279	730
Hip circumference (cm)	Frequent vs rare	1.84 (−0.59 to 4.27)	0.137	0.412	465	0.89 (−1.00 to 2.77)	0.355	0.557	730
Waist–hip ratio	Frequent vs rare	0.0087 (−0.0067 to 0.0240)	0.267	0.540	465	0.0123 (0.0003 to 0.0242)	0.044	0.237	730
Visceral fat (%)	Frequent vs rare	1.11 (−1.80 to 4.02)	0.452	0.636	334	1.00 (−1.33 to 3.34)	0.400	0.575	487
Fasting glucose (mg/dL)	Frequent vs rare	0.80 (−1.54 to 3.14)	0.503	0.661	429	1.41 (−0.63 to 3.45)	0.176	0.391	647
2-h glucose (mg/dL)	Frequent vs rare	3.48 (−3.35 to 10.31)	0.317	0.551	427	2.85 (−2.73 to 8.42)	0.316	0.507	640
Glucose AUC (scaled 0–1)	Frequent vs rare	0.0164 (−0.0024 to 0.0351)	0.087	0.315	419	0.0151 (−0.0002 to 0.0304)	0.053	0.237	629
HbA1c (%)	Frequent vs rare	0.06 (−0.04 to 0.16)	0.210	0.498	429	0.08 (−0.01 to 0.17)	0.099	0.298	646
Fasting insulin (μIU/mL)	Frequent vs rare	−0.70 (−2.42 to 1.03)	0.426	0.636	429	−0.51 (−2.06 to 1.03)	0.515	0.696	648
Insulin AUC (scaled 0–1)	Frequent vs rare	0.0162 (−0.0079 to 0.0403)	0.186	0.476	416	0.0081 (−0.0102 to 0.0263)	0.386	0.567	625
HOMA-IR	Frequent vs rare	−0.17 (−0.61 to 0.27)	0.438	0.636	429	−0.09 (−0.49 to 0.31)	0.668	0.794	647
HOMA-B	Frequent vs rare	−21.39 (−55.91 to 13.14)	0.224	0.499	428	−17.36 (−43.53 to 8.80)	0.193	0.392	646
Total cholesterol (mg/dL)	Frequent vs rare	−2.38 (−10.47 to 5.72)	0.564	0.684	436	−4.15 (−10.68 to 2.38)	0.212	0.396	679
LDL cholesterol (mg/dL)	Frequent vs rare	−2.00 (−9.51 to 5.52)	0.602	0.707	436	−4.05 (−10.05 to 1.95)	0.185	0.391	679

Outcome	Contrast	Primary model: age, sex, daily energy intake, physical inactivity				Sensitivity model: age, sex, physical inactivity (no energy intake)			
		**β or OR (95% CI)**	**p**	**q**	**n**	**β or OR (95% CI)**	**p**	**q**	**n**
HDL cholesterol (mg/dL)	Frequent vs rare	−2.71 (−5.45 to 0.04)	0.053	0.262	436	−1.39 (−3.68 to 0.91)	0.237	0.411	680
Triglycerides (mg/dL)	Frequent vs rare	11.64 (−1.50 to 24.78)	0.082	0.315	436	6.26 (−4.15 to 16.68)	0.238	0.411	681
Prevalent T2DM	Frequent vs rare	OR 0.96 (0.42 to 2.28)	0.916	0.916	465 (33)	OR 1.07 (0.62 to 1.90)	0.804	0.880	735 (83)

Estimates are regression coefficients (β, in the units of the outcome) for continuous outcomes and odds ratios (OR) for prevalent type 2 diabetes (T2DM); for T2DM, n is the number of participants with the number of cases in parentheses. Reference categories: never coffee consumption and rare snacking.

Primary model: adjusted for age, sex, daily energy intake, and physical inactivity, as specified in Section 2.3; it is restricted to participants with energy-intake data from 3-day dietary records. Sensitivity model: adjusted for age, sex, and physical inactivity without energy intake, which allows all participants with questionnaire and outcome data to be included. Each model used all participants with complete data for its variables (complete-case analysis). Coffee consumption and snacking frequency were modelled separately, so n can differ slightly between them (4 participants completed only the snacking questionnaire and 1 only the coffee questionnaire).

q, Benjamini–Hochberg false discovery rate-adjusted p-value across the 69 tests of each model. Glucose and insulin area under the curve (AUC) were derived from the 75 g oral glucose tolerance test and min–max scaled to a 0–1 range (Section 2.2); glycaemic measurements were not available for participants with diagnosed T2DM.

BMI, body mass index; CI, confidence interval; HbA1c, glycated haemoglobin; HDL, high-density lipoprotein; HOMA-B, homeostatic model assessment of β-cell function; HOMA-IR, homeostatic model assessment of insulin resistance; LDL, low-density lipoprotein.

Table S2. Gene × behaviour interaction estimates for BDNF and TCF7L2 variants.

Variant	Interaction term	Outcome	n	β or OR (95% CI)	p (term)	p (joint test)	q (joint test)
BDNF rs10501087	Weekly coffee × allele	Body weight (kg)	451	0.79 (−6.47 to 8.04)	0.832	0.966	0.989
BDNF rs10501087	Daily coffee × allele	Body weight (kg)	451	1.04 (−10.01 to 12.09)	0.853	0.966	0.989
BDNF rs10501087	Weekly coffee × allele	BMI (kg/m²)	451	0.67 (−1.74 to 3.09)	0.584	0.713	0.946
BDNF rs10501087	Daily coffee × allele	BMI (kg/m²)	451	1.30 (−2.39 to 4.98)	0.490	0.713	0.946
BDNF rs10501087	Weekly coffee × allele	Fat mass (kg)	449	1.51 (−3.54 to 6.55)	0.558	0.840	0.962
BDNF rs10501087	Daily coffee × allele	Fat mass (kg)	449	0.57 (−7.15 to 8.29)	0.885	0.840	0.962
BDNF rs10501087	Weekly coffee × allele	Body fat (%)	449	2.39 (−0.77 to 5.55)	0.137	0.315	0.804
BDNF rs10501087	Daily coffee × allele	Body fat (%)	449	−0.22 (−5.05 to 4.62)	0.930	0.315	0.804
BDNF rs10501087	Weekly coffee × allele	Fat-free mass (kg)	449	−0.88 (−3.95 to 2.18)	0.572	0.739	0.946
BDNF rs10501087	Daily coffee × allele	Fat-free mass (kg)	449	1.06 (−3.64 to 5.75)	0.659	0.739	0.946
BDNF rs10501087	Weekly coffee × allele	Muscle mass (kg)	325	−0.55 (−2.15 to 1.05)	0.498	0.672	0.946
BDNF rs10501087	Daily coffee × allele	Muscle mass (kg)	325	0.53 (−1.67 to 2.73)	0.637	0.672	0.946
BDNF rs10501087	Weekly coffee × allele	Waist circumference (cm)	451	1.09 (−4.79 to 6.98)	0.716	0.919	0.976
BDNF rs10501087	Daily coffee × allele	Waist circumference (cm)	451	1.11 (−7.86 to 10.09)	0.807	0.919	0.976
BDNF rs10501087	Weekly coffee × allele	Hip circumference (cm)	451	2.77 (−1.92 to 7.46)	0.246	0.391	0.890
BDNF rs10501087	Daily coffee × allele	Hip circumference (cm)	451	−1.98 (−9.13 to 5.17)	0.586	0.391	0.890
BDNF rs10501087	Weekly coffee × allele	Waist–hip ratio	451	−0.0127 (−0.0422 to 0.0169)	0.400	0.213	0.725
BDNF rs10501087	Daily coffee × allele	Waist–hip ratio	451	0.0321 (−0.0129 to 0.0772)	0.162	0.213	0.725
BDNF rs10501087	Weekly coffee × allele	Visceral fat (%)	325	−0.90 (−6.72 to 4.92)	0.760	0.733	0.946
BDNF rs10501087	Daily coffee × allele	Visceral fat (%)	325	2.74 (−5.25 to 10.73)	0.501	0.733	0.946
BDNF rs10501087	Weekly coffee × allele	Fasting glucose (mg/dL)	421	1.95 (−2.43 to 6.34)	0.382	0.649	0.946
BDNF rs10501087	Daily coffee × allele	Fasting glucose (mg/dL)	421	1.57 (−5.38 to 8.53)	0.657	0.649	0.946
BDNF rs10501087	Weekly coffee × allele	2-h glucose (mg/dL)	419	3.35 (−9.35 to 16.06)	0.604	0.347	0.863
BDNF rs10501087	Daily coffee × allele	2-h glucose (mg/dL)	419	14.58 (−5.55 to 34.70)	0.155	0.347	0.863
BDNF rs10501087	Weekly coffee × allele	Glucose AUC (scaled 0–1)	411	0.0003 (−0.0356 to 0.0362)	0.987	0.622	0.946
BDNF rs10501087	Daily coffee × allele	Glucose AUC (scaled 0–1)	411	0.0271 (−0.0279 to 0.0821)	0.334	0.622	0.946
BDNF rs10501087	Weekly coffee × allele	HbA1c (%)	421	−0.04 (−0.22 to 0.14)	0.647	0.253	0.725
BDNF rs10501087	Daily coffee × allele	HbA1c (%)	421	0.22 (−0.07 to 0.50)	0.132	0.253	0.725
BDNF rs10501087	Weekly coffee × allele	Fasting insulin (µIU/mL)	421	−1.26 (−4.49 to 1.97)	0.443	0.730	0.946
BDNF rs10501087	Daily coffee × allele	Fasting insulin (µIU/mL)	421	0.22 (−4.91 to 5.34)	0.934	0.730	0.946
BDNF rs10501087	Weekly coffee × allele	Insulin AUC (scaled 0–1)	408	−0.0184 (−0.0645 to 0.0277)	0.433	0.728	0.946
BDNF rs10501087	Daily coffee × allele	Insulin AUC (scaled 0–1)	408	−0.0090 (−0.0795 to 0.0616)	0.803	0.728	0.946
BDNF rs10501087	Weekly coffee × allele	HOMA-IR	421	−0.31 (−1.13 to 0.51)	0.459	0.724	0.946
BDNF rs10501087	Daily coffee × allele	HOMA-IR	421	0.13 (−1.17 to 1.43)	0.845	0.724	0.946
BDNF rs10501087	Weekly coffee × allele	HOMA-B	420	−43.76 (−108.48 to 20.97)	0.185	0.368	0.878
BDNF rs10501087	Daily coffee × allele	HOMA-B	420	−35.70 (−138.39 to 67.00)	0.495	0.368	0.878

Variant	Interaction term	Outcome	n	β or OR (95% CI)	p (term)	p (joint test)	q (joint test)
BDNF rs10501087	Weekly coffee × allele	Total cholesterol (mg/dL)	423	9.92 (−5.87 to 25.71)	0.218	0.463	0.945
BDNF rs10501087	Daily coffee × allele	Total cholesterol (mg/dL)	423	3.81 (−19.58 to 27.21)	0.749	0.463	0.945
BDNF rs10501087	Weekly coffee × allele	LDL cholesterol (mg/dL)	423	13.45 (−1.20 to 28.10)	0.072	0.194	0.725
BDNF rs10501087	Daily coffee × allele	LDL cholesterol (mg/dL)	423	5.10 (−16.60 to 26.79)	0.644	0.194	0.725
BDNF rs10501087	Weekly coffee × allele	HDL cholesterol (mg/dL)	423	−2.92 (−8.26 to 2.42)	0.283	0.149	0.710
BDNF rs10501087	Daily coffee × allele	HDL cholesterol (mg/dL)	423	5.85 (−2.07 to 13.76)	0.147	0.149	0.710
BDNF rs10501087	Weekly coffee × allele	Triglycerides (mg/dL)	423	−3.05 (−28.88 to 22.77)	0.816	0.188	0.725
BDNF rs10501087	Daily coffee × allele	Triglycerides (mg/dL)	423	−35.65 (−73.91 to 2.60)	0.068	0.188	0.725
BDNF rs10501087	Weekly coffee × allele	Prevalent T2DM	451 (27)	OR 2.31 (0.09 to 26.26)	0.536	0.839	0.962
BDNF rs10501087	Daily coffee × allele	Prevalent T2DM	451 (27)	OR 1.10 (0.04 to 12.60)	0.942	0.839	0.962
BDNF rs10501087	Frequent snacking × allele	Body weight (kg)	452	−3.50 (−9.61 to 2.61)	0.261	0.261	0.725
BDNF rs10501087	Frequent snacking × allele	BMI (kg/m²)	452	−1.67 (−3.70 to 0.37)	0.109	0.109	0.710
BDNF rs10501087	Frequent snacking × allele	Fat mass (kg)	450	−2.35 (−6.61 to 1.91)	0.279	0.279	0.755
BDNF rs10501087	Frequent snacking × allele	Body fat (%)	450	−1.77 (−4.44 to 0.89)	0.192	0.192	0.725
BDNF rs10501087	Frequent snacking × allele	Fat-free mass (kg)	450	−1.51 (−4.10 to 1.09)	0.254	0.254	0.725
BDNF rs10501087	Frequent snacking × allele	Muscle mass (kg)	326	−0.75 (−2.03 to 0.53)	0.252	0.252	0.725
BDNF rs10501087	Frequent snacking × allele	Waist circumference (cm)	452	−4.59 (−9.54 to 0.36)	0.069	0.069	0.710
BDNF rs10501087	Frequent snacking × allele	Hip circumference (cm)	452	−3.17 (−7.14 to 0.79)	0.116	0.116	0.710
BDNF rs10501087	Frequent snacking × allele	Waist–hip ratio	452	−0.0149 (−0.0399 to 0.0102)	0.244	0.244	0.725
BDNF rs10501087	Frequent snacking × allele	Visceral fat (%)	326	−1.30 (−5.99 to 3.39)	0.586	0.586	0.946
BDNF rs10501087	Frequent snacking × allele	Fasting glucose (mg/dL)	422	1.37 (−2.37 to 5.11)	0.472	0.472	0.945
BDNF rs10501087	Frequent snacking × allele	2-h glucose (mg/dL)	420	−4.65 (−15.65 to 6.35)	0.406	0.406	0.890
BDNF rs10501087	Frequent snacking × allele	Glucose AUC (scaled 0–1)	412	−0.0235 (−0.0538 to 0.0068)	0.127	0.127	0.710
BDNF rs10501087	Frequent snacking × allele	HbA1c (%)	422	−0.12 (−0.27 to 0.03)	0.119	0.119	0.710
BDNF rs10501087	Frequent snacking × allele	Fasting insulin (μIU/mL)	422	0.24 (−2.53 to 3.01)	0.863	0.863	0.962
BDNF rs10501087	Frequent snacking × allele	Insulin AUC (scaled 0–1)	409	−0.0192 (−0.0582 to 0.0198)	0.335	0.335	0.844
BDNF rs10501087	Frequent snacking × allele	HOMA-IR	422	0.10 (−0.61 to 0.80)	0.791	0.791	0.961
BDNF rs10501087	Frequent snacking × allele	HOMA-B	421	−62.79 (−117.76 to −7.82)	0.025	0.025	0.594
BDNF rs10501087	Frequent snacking × allele	Total cholesterol (mg/dL)	424	−9.85 (−23.17 to 3.47)	0.147	0.147	0.710
BDNF rs10501087	Frequent snacking × allele	LDL cholesterol (mg/dL)	424	−7.05 (−19.43 to 5.34)	0.264	0.264	0.725
BDNF rs10501087	Frequent snacking × allele	HDL cholesterol (mg/dL)	424	−3.23 (−7.77 to 1.30)	0.162	0.162	0.710
BDNF rs10501087	Frequent snacking × allele	Triglycerides (mg/dL)	424	2.15 (−19.69 to 23.98)	0.847	0.847	0.962
BDNF rs10501087	Frequent snacking × allele	Prevalent T2DM	452 (27)	OR 0.92 (0.15 to 7.50)	0.928	0.929	0.976
TCF7L2 rs4506565	Weekly coffee × allele	Body weight (kg)	456	−0.66 (−7.63 to 6.31)	0.851	0.936	0.978
TCF7L2 rs4506565	Daily coffee × allele	Body weight (kg)	456	1.18 (−7.38 to 9.74)	0.787	0.936	0.978
TCF7L2 rs4506565	Weekly coffee × allele	BMI (kg/m²)	456	−0.24 (−2.57 to 2.08)	0.836	0.872	0.962
TCF7L2 rs4506565	Daily coffee × allele	BMI (kg/m²)	456	0.63 (−2.22 to 3.48)	0.666	0.872	0.962

Variant	Interaction term	Outcome	n	β or OR (95% CI)	p (term)	p (joint test)	q (joint test)
TCF7L2 rs4506565	Weekly coffee × allele	Fat mass (kg)	454	1.11 (−3.72 to 5.95)	0.652	0.833	0.962
TCF7L2 rs4506565	Daily coffee × allele	Fat mass (kg)	454	1.46 (−4.52 to 7.44)	0.631	0.833	0.962
TCF7L2 rs4506565	Weekly coffee × allele	Body fat (%)	454	1.48 (−1.54 to 4.49)	0.337	0.628	0.946
TCF7L2 rs4506565	Daily coffee × allele	Body fat (%)	454	0.23 (−3.50 to 3.96)	0.905	0.628	0.946
TCF7L2 rs4506565	Weekly coffee × allele	Fat-free mass (kg)	454	−1.62 (−4.56 to 1.32)	0.280	0.546	0.946
TCF7L2 rs4506565	Daily coffee × allele	Fat-free mass (kg)	454	−0.77 (−4.40 to 2.86)	0.678	0.546	0.946
TCF7L2 rs4506565	Weekly coffee × allele	Muscle mass (kg)	328	0.18 (−1.28 to 1.64)	0.809	0.787	0.961
TCF7L2 rs4506565	Daily coffee × allele	Muscle mass (kg)	328	0.64 (−1.21 to 2.49)	0.496	0.787	0.961
TCF7L2 rs4506565	Weekly coffee × allele	Waist circumference (cm)	456	−1.65 (−7.28 to 3.98)	0.565	0.717	0.946
TCF7L2 rs4506565	Daily coffee × allele	Waist circumference (cm)	456	1.60 (−5.32 to 8.52)	0.649	0.717	0.946
TCF7L2 rs4506565	Weekly coffee × allele	Hip circumference (cm)	456	−1.25 (−5.74 to 3.25)	0.586	0.620	0.946
TCF7L2 rs4506565	Daily coffee × allele	Hip circumference (cm)	456	1.94 (−3.58 to 7.46)	0.490	0.620	0.946
TCF7L2 rs4506565	Weekly coffee × allele	Waist–hip ratio	456	−0.0077 (−0.0361 to 0.0206)	0.593	0.842	0.962
TCF7L2 rs4506565	Daily coffee × allele	Waist–hip ratio	456	−0.0060 (−0.0408 to 0.0289)	0.737	0.842	0.962
TCF7L2 rs4506565	Weekly coffee × allele	Visceral fat (%)	328	2.00 (−3.25 to 7.25)	0.455	0.079	0.710
TCF7L2 rs4506565	Daily coffee × allele	Visceral fat (%)	328	−6.62 (−13.28 to 0.04)	0.051	0.079	0.710
TCF7L2 rs4506565	Weekly coffee × allele	Fasting glucose (mg/dL)	420	2.03 (−2.29 to 6.35)	0.357	0.647	0.946
TCF7L2 rs4506565	Daily coffee × allele	Fasting glucose (mg/dL)	420	0.05 (−5.59 to 5.68)	0.986	0.647	0.946
TCF7L2 rs4506565	Weekly coffee × allele	2-h glucose (mg/dL)	418	−0.08 (−12.58 to 12.41)	0.990	0.860	0.962
TCF7L2 rs4506565	Daily coffee × allele	2-h glucose (mg/dL)	418	4.45 (−11.84 to 20.73)	0.592	0.860	0.962
TCF7L2 rs4506565	Weekly coffee × allele	Glucose AUC (scaled 0–1)	410	−0.0078 (−0.0437 to 0.0281)	0.668	0.911	0.976
TCF7L2 rs4506565	Daily coffee × allele	Glucose AUC (scaled 0–1)	410	−0.0006 (−0.0453 to 0.0440)	0.978	0.911	0.976
TCF7L2 rs4506565	Weekly coffee × allele	HbA1c (%)	420	0.11 (−0.06 to 0.29)	0.203	0.405	0.890
TCF7L2 rs4506565	Daily coffee × allele	HbA1c (%)	420	0.08 (−0.15 to 0.31)	0.513	0.405	0.890
TCF7L2 rs4506565	Weekly coffee × allele	Fasting insulin (μIU/mL)	420	−3.08 (−6.26 to 0.09)	0.057	0.161	0.710
TCF7L2 rs4506565	Daily coffee × allele	Fasting insulin (μIU/mL)	420	−1.01 (−5.15 to 3.12)	0.630	0.161	0.710
TCF7L2 rs4506565	Weekly coffee × allele	Insulin AUC (scaled 0–1)	407	−0.0211 (−0.0670 to 0.0248)	0.367	0.157	0.710
TCF7L2 rs4506565	Daily coffee × allele	Insulin AUC (scaled 0–1)	407	−0.0533 (−0.1104 to 0.0038)	0.067	0.157	0.710
TCF7L2 rs4506565	Weekly coffee × allele	HOMA-IR	420	−0.76 (−1.57 to 0.05)	0.064	0.179	0.725
TCF7L2 rs4506565	Daily coffee × allele	HOMA-IR	420	−0.13 (−1.19 to 0.92)	0.805	0.179	0.725
TCF7L2 rs4506565	Weekly coffee × allele	HOMA-B	419	−44.58 (−108.57 to 19.42)	0.172	0.385	0.890
TCF7L2 rs4506565	Daily coffee × allele	HOMA-B	419	−2.30 (−85.74 to 81.15)	0.957	0.385	0.890
TCF7L2 rs4506565	Weekly coffee × allele	Total cholesterol (mg/dL)	428	−1.84 (−16.95 to 13.28)	0.811	0.951	0.984
TCF7L2 rs4506565	Daily coffee × allele	Total cholesterol (mg/dL)	428	1.42 (−16.51 to 19.36)	0.876	0.951	0.984
TCF7L2 rs4506565	Weekly coffee × allele	LDL cholesterol (mg/dL)	428	−5.12 (−19.12 to 8.88)	0.473	0.748	0.946
TCF7L2 rs4506565	Daily coffee × allele	LDL cholesterol (mg/dL)	428	−3.31 (−19.92 to 13.30)	0.696	0.748	0.946
TCF7L2 rs4506565	Weekly coffee × allele	HDL cholesterol (mg/dL)	428	2.75 (−2.35 to 7.86)	0.290	0.560	0.946
TCF7L2 rs4506565	Daily coffee × allele	HDL cholesterol (mg/dL)	428	1.21 (−4.85 to 7.27)	0.695	0.560	0.946

Variant	Interaction term	Outcome	n	β or OR (95% CI)	p (term)	p (joint test)	q (joint test)
TCF7L2 rs4506565	Weekly coffee × allele	Triglycerides (mg/dL)	428	2.65 (−21.94 to 27.23)	0.832	0.495	0.946
TCF7L2 rs4506565	Daily coffee × allele	Triglycerides (mg/dL)	428	17.62 (−11.56 to 46.79)	0.236	0.495	0.946
TCF7L2 rs4506565	Weekly coffee × allele	Prevalent T2DM	456 (33)	OR 1.05 (0.16 to 6.44)	0.953	0.424	0.918
TCF7L2 rs4506565	Daily coffee × allele	Prevalent T2DM	456 (33)	OR 0.32 (0.04 to 1.81)	0.223	0.424	0.918
TCF7L2 rs4506565	Frequent snacking × allele	Body weight (kg)	457	−1.54 (−7.42 to 4.35)	0.608	0.608	0.946
TCF7L2 rs4506565	Frequent snacking × allele	BMI (kg/m²)	457	−0.55 (−2.51 to 1.41)	0.581	0.581	0.946
TCF7L2 rs4506565	Frequent snacking × allele	Fat mass (kg)	455	−1.83 (−5.92 to 2.25)	0.379	0.379	0.890
TCF7L2 rs4506565	Frequent snacking × allele	Body fat (%)	455	−1.67 (−4.21 to 0.87)	0.198	0.198	0.725
TCF7L2 rs4506565	Frequent snacking × allele	Fat-free mass (kg)	455	0.53 (−1.96 to 3.02)	0.675	0.675	0.946
TCF7L2 rs4506565	Frequent snacking × allele	Muscle mass (kg)	329	0.39 (−0.84 to 1.62)	0.530	0.530	0.946
TCF7L2 rs4506565	Frequent snacking × allele	Waist circumference (cm)	457	−1.28 (−6.04 to 3.48)	0.598	0.598	0.946
TCF7L2 rs4506565	Frequent snacking × allele	Hip circumference (cm)	457	1.13 (−2.68 to 4.94)	0.561	0.561	0.946
TCF7L2 rs4506565	Frequent snacking × allele	Waist–hip ratio	457	−0.0220 (−0.0459 to 0.0019)	0.071	0.071	0.710
TCF7L2 rs4506565	Frequent snacking × allele	Visceral fat (%)	329	−3.68 (−8.14 to 0.78)	0.106	0.106	0.710
TCF7L2 rs4506565	Frequent snacking × allele	Fasting glucose (mg/dL)	421	−4.24 (−7.90 to −0.58)	0.023	0.023	0.594
TCF7L2 rs4506565	Frequent snacking × allele	2-h glucose (mg/dL)	419	−1.23 (−11.97 to 9.52)	0.822	0.822	0.962
TCF7L2 rs4506565	Frequent snacking × allele	Glucose AUC (scaled 0–1)	411	−0.0171 (−0.0469 to 0.0128)	0.261	0.261	0.725
TCF7L2 rs4506565	Frequent snacking × allele	HbA1c (%)	421	0.03 (−0.12 to 0.18)	0.673	0.673	0.946
TCF7L2 rs4506565	Frequent snacking × allele	Fasting insulin (μIU/mL)	421	−1.56 (−4.29 to 1.16)	0.260	0.260	0.725
TCF7L2 rs4506565	Frequent snacking × allele	Insulin AUC (scaled 0–1)	408	−0.0223 (−0.0607 to 0.0161)	0.255	0.255	0.725
TCF7L2 rs4506565	Frequent snacking × allele	HOMA-IR	421	−0.50 (−1.19 to 0.19)	0.157	0.157	0.710
TCF7L2 rs4506565	Frequent snacking × allele	HOMA-B	420	21.66 (−32.81 to 76.13)	0.435	0.435	0.920
TCF7L2 rs4506565	Frequent snacking × allele	Total cholesterol (mg/dL)	429	−2.54 (−15.21 to 10.13)	0.694	0.694	0.946
TCF7L2 rs4506565	Frequent snacking × allele	LDL cholesterol (mg/dL)	429	−1.35 (−13.09 to 10.39)	0.821	0.821	0.962
TCF7L2 rs4506565	Frequent snacking × allele	HDL cholesterol (mg/dL)	429	4.43 (0.16 to 8.69)	0.042	0.042	0.710
TCF7L2 rs4506565	Frequent snacking × allele	Triglycerides (mg/dL)	429	−28.08 (−48.46 to −7.69)	0.007	0.007	0.496
TCF7L2 rs4506565	Frequent snacking × allele	Prevalent T2DM	457 (33)	OR 2.29 (0.61 to 9.52)	0.228	0.220	0.725
TCF7L2 rs7901695	Weekly coffee × allele	Body weight (kg)	456	−0.84 (−7.80 to 6.12)	0.812	0.952	0.984
TCF7L2 rs7901695	Daily coffee × allele	Body weight (kg)	456	0.67 (−7.90 to 9.24)	0.878	0.952	0.984
TCF7L2 rs7901695	Weekly coffee × allele	BMI (kg/m²)	456	−0.30 (−2.62 to 2.01)	0.796	0.927	0.976
TCF7L2 rs7901695	Daily coffee × allele	BMI (kg/m²)	456	0.34 (−2.51 to 3.19)	0.815	0.927	0.976
TCF7L2 rs7901695	Weekly coffee × allele	Fat mass (kg)	454	0.79 (−4.04 to 5.63)	0.747	0.920	0.976
TCF7L2 rs7901695	Daily coffee × allele	Fat mass (kg)	454	0.94 (−5.04 to 6.93)	0.757	0.920	0.976
TCF7L2 rs7901695	Weekly coffee × allele	Body fat (%)	454	1.09 (−1.93 to 4.12)	0.478	0.760	0.946
TCF7L2 rs7901695	Daily coffee × allele	Body fat (%)	454	−0.13 (−3.87 to 3.62)	0.947	0.760	0.946
TCF7L2 rs7901695	Weekly coffee × allele	Fat-free mass (kg)	454	−1.49 (−4.44 to 1.45)	0.320	0.596	0.946
TCF7L2 rs7901695	Daily coffee × allele	Fat-free mass (kg)	454	−0.76 (−4.41 to 2.89)	0.682	0.596	0.946

Variant	Interaction term	Outcome	n	β or OR (95% CI)	p (term)	p (joint test)	q (joint test)
TCF7L2 rs7901695	Weekly coffee × allele	Muscle mass (kg)	326	−0.03 (−1.50 to 1.43)	0.966	0.794	0.961
TCF7L2 rs7901695	Daily coffee × allele	Muscle mass (kg)	326	0.62 (−1.23 to 2.47)	0.511	0.794	0.961
TCF7L2 rs7901695	Weekly coffee × allele	Waist circumference (cm)	456	−2.09 (−7.69 to 3.52)	0.465	0.675	0.946
TCF7L2 rs7901695	Daily coffee × allele	Waist circumference (cm)	456	1.22 (−5.69 to 8.12)	0.729	0.675	0.946
TCF7L2 rs7901695	Weekly coffee × allele	Hip circumference (cm)	456	−1.39 (−5.87 to 3.09)	0.541	0.642	0.946
TCF7L2 rs7901695	Daily coffee × allele	Hip circumference (cm)	456	1.63 (−3.89 to 7.14)	0.562	0.642	0.946
TCF7L2 rs7901695	Weekly coffee × allele	Waist–hip ratio	456	−0.0106 (−0.0387 to 0.0175)	0.458	0.739	0.946
TCF7L2 rs7901695	Daily coffee × allele	Waist–hip ratio	456	−0.0066 (−0.0412 to 0.0279)	0.706	0.739	0.946
TCF7L2 rs7901695	Weekly coffee × allele	Visceral fat (%)	326	3.12 (−2.14 to 8.38)	0.244	0.032	0.652
TCF7L2 rs7901695	Daily coffee × allele	Visceral fat (%)	326	−7.07 (−13.72 to −0.43)	0.037	0.032	0.652
TCF7L2 rs7901695	Weekly coffee × allele	Fasting glucose (mg/dL)	420	1.79 (−2.55 to 6.12)	0.419	0.680	0.946
TCF7L2 rs7901695	Daily coffee × allele	Fasting glucose (mg/dL)	420	−0.54 (−6.20 to 5.13)	0.853	0.680	0.946
TCF7L2 rs7901695	Weekly coffee × allele	2-h glucose (mg/dL)	418	0.15 (−12.34 to 12.65)	0.981	0.853	0.962
TCF7L2 rs7901695	Daily coffee × allele	2-h glucose (mg/dL)	418	4.63 (−11.68 to 20.94)	0.577	0.853	0.962
TCF7L2 rs7901695	Weekly coffee × allele	Glucose AUC (scaled 0–1)	410	−0.0100 (−0.0458 to 0.0258)	0.584	0.861	0.962
TCF7L2 rs7901695	Daily coffee × allele	Glucose AUC (scaled 0–1)	410	−0.0023 (−0.0468 to 0.0423)	0.920	0.861	0.962
TCF7L2 rs7901695	Weekly coffee × allele	HbA1c (%)	420	0.11 (−0.06 to 0.29)	0.209	0.406	0.890
TCF7L2 rs7901695	Daily coffee × allele	HbA1c (%)	420	0.08 (−0.15 to 0.31)	0.485	0.406	0.890
TCF7L2 rs7901695	Weekly coffee × allele	Fasting insulin (μIU/mL)	420	−3.38 (−6.53 to −0.22)	0.036	0.109	0.710
TCF7L2 rs7901695	Daily coffee × allele	Fasting insulin (μIU/mL)	420	−1.15 (−5.27 to 2.97)	0.584	0.109	0.710
TCF7L2 rs7901695	Weekly coffee × allele	Insulin AUC (scaled 0–1)	407	−0.0198 (−0.0656 to 0.0260)	0.396	0.115	0.710
TCF7L2 rs7901695	Daily coffee × allele	Insulin AUC (scaled 0–1)	407	−0.0586 (−0.1156 to −0.0017)	0.044	0.115	0.710
TCF7L2 rs7901695	Weekly coffee × allele	HOMA-IR	420	−0.84 (−1.64 to −0.04)	0.041	0.123	0.710
TCF7L2 rs7901695	Daily coffee × allele	HOMA-IR	420	−0.19 (−1.24 to 0.86)	0.724	0.123	0.710
TCF7L2 rs7901695	Weekly coffee × allele	HOMA-B	419	−45.48 (−109.29 to 18.32)	0.162	0.366	0.878
TCF7L2 rs7901695	Daily coffee × allele	HOMA-B	419	−1.44 (−84.76 to 81.89)	0.973	0.366	0.878
TCF7L2 rs7901695	Weekly coffee × allele	Total cholesterol (mg/dL)	429	0.52 (−14.55 to 15.58)	0.946	0.989	0.989
TCF7L2 rs7901695	Daily coffee × allele	Total cholesterol (mg/dL)	429	1.30 (−16.62 to 19.23)	0.886	0.989	0.989
TCF7L2 rs7901695	Weekly coffee × allele	LDL cholesterol (mg/dL)	429	−1.64 (−15.63 to 12.35)	0.818	0.907	0.976
TCF7L2 rs7901695	Daily coffee × allele	LDL cholesterol (mg/dL)	429	−3.51 (−20.16 to 13.14)	0.679	0.907	0.976
TCF7L2 rs7901695	Weekly coffee × allele	HDL cholesterol (mg/dL)	429	2.16 (−2.93 to 7.26)	0.404	0.679	0.946
TCF7L2 rs7901695	Daily coffee × allele	HDL cholesterol (mg/dL)	429	1.35 (−4.71 to 7.42)	0.661	0.679	0.946
TCF7L2 rs7901695	Weekly coffee × allele	Triglycerides (mg/dL)	429	−0.05 (−24.58 to 24.49)	0.997	0.493	0.946
TCF7L2 rs7901695	Daily coffee × allele	Triglycerides (mg/dL)	429	17.32 (−11.88 to 46.51)	0.244	0.493	0.946
TCF7L2 rs7901695	Weekly coffee × allele	Prevalent T2DM	456 (33)	OR 0.93 (0.14 to 5.92)	0.939	0.260	0.725
TCF7L2 rs7901695	Daily coffee × allele	Prevalent T2DM	456 (33)	OR 0.22 (0.03 to 1.35)	0.123	0.260	0.725
TCF7L2 rs7901695	Frequent snacking × allele	Body weight (kg)	457	−1.94 (−7.83 to 3.95)	0.518	0.518	0.946
TCF7L2 rs7901695	Frequent snacking × allele	BMI (kg/m²)	457	−0.65 (−2.61 to 1.31)	0.515	0.515	0.946

Variant	Interaction term	Outcome	n	β or OR (95% CI)	p (term)	p (joint test)	q (joint test)
TCF7L2 rs7901695	Frequent snacking × allele	Fat mass (kg)	455	−2.14 (−6.23 to 1.95)	0.304	0.304	0.798
TCF7L2 rs7901695	Frequent snacking × allele	Body fat (%)	455	−1.91 (−4.46 to 0.64)	0.142	0.142	0.710
TCF7L2 rs7901695	Frequent snacking × allele	Fat-free mass (kg)	455	0.44 (−2.07 to 2.94)	0.732	0.732	0.946
TCF7L2 rs7901695	Frequent snacking × allele	Muscle mass (kg)	327	0.25 (−0.98 to 1.48)	0.687	0.687	0.946
TCF7L2 rs7901695	Frequent snacking × allele	Waist circumference (cm)	457	−1.59 (−6.34 to 3.16)	0.511	0.511	0.946
TCF7L2 rs7901695	Frequent snacking × allele	Hip circumference (cm)	457	0.80 (−3.01 to 4.60)	0.681	0.681	0.946
TCF7L2 rs7901695	Frequent snacking × allele	Waist–hip ratio	457	−0.0222 (−0.0459 to 0.0015)	0.066	0.066	0.710
TCF7L2 rs7901695	Frequent snacking × allele	Visceral fat (%)	327	−3.30 (−7.78 to 1.18)	0.148	0.148	0.710
TCF7L2 rs7901695	Frequent snacking × allele	Fasting glucose (mg/dL)	421	−4.45 (−8.13 to −0.78)	0.018	0.018	0.594
TCF7L2 rs7901695	Frequent snacking × allele	2-h glucose (mg/dL)	419	−1.25 (−12.04 to 9.53)	0.819	0.819	0.962
TCF7L2 rs7901695	Frequent snacking × allele	Glucose AUC (scaled 0–1)	411	−0.0187 (−0.0485 to 0.0112)	0.219	0.219	0.725
TCF7L2 rs7901695	Frequent snacking × allele	HbA1c (%)	421	0.03 (−0.12 to 0.18)	0.673	0.673	0.946
TCF7L2 rs7901695	Frequent snacking × allele	Fasting insulin (μIU/mL)	421	−1.70 (−4.41 to 1.02)	0.221	0.221	0.725
TCF7L2 rs7901695	Frequent snacking × allele	Insulin AUC (scaled 0–1)	408	−0.0228 (−0.0612 to 0.0156)	0.243	0.243	0.725
TCF7L2 rs7901695	Frequent snacking × allele	HOMA-IR	421	−0.54 (−1.23 to 0.16)	0.129	0.129	0.710
TCF7L2 rs7901695	Frequent snacking × allele	HOMA-B	420	21.67 (−32.82 to 76.16)	0.435	0.435	0.920
TCF7L2 rs7901695	Frequent snacking × allele	Total cholesterol (mg/dL)	430	−1.84 (−14.50 to 10.82)	0.775	0.775	0.957
TCF7L2 rs7901695	Frequent snacking × allele	LDL cholesterol (mg/dL)	430	−0.10 (−11.85 to 11.65)	0.987	0.987	0.989
TCF7L2 rs7901695	Frequent snacking × allele	HDL cholesterol (mg/dL)	430	3.88 (−0.38 to 8.15)	0.074	0.074	0.710
TCF7L2 rs7901695	Frequent snacking × allele	Triglycerides (mg/dL)	430	−28.12 (−48.50 to −7.73)	0.007	0.007	0.496
TCF7L2 rs7901695	Frequent snacking × allele	Prevalent T2DM	457 (33)	OR 2.05 (0.55 to 8.47)	0.299	0.291	0.777
TCF7L2 rs7903146	Weekly coffee × allele	Body weight (kg)	460	−1.00 (−7.68 to 5.68)	0.769	0.882	0.966
TCF7L2 rs7903146	Daily coffee × allele	Body weight (kg)	460	1.33 (−6.62 to 9.28)	0.743	0.882	0.966
TCF7L2 rs7903146	Weekly coffee × allele	BMI (kg/m²)	460	−0.37 (−2.59 to 1.86)	0.746	0.842	0.962
TCF7L2 rs7903146	Daily coffee × allele	BMI (kg/m²)	460	0.54 (−2.11 to 3.19)	0.687	0.842	0.962
TCF7L2 rs7903146	Weekly coffee × allele	Fat mass (kg)	458	0.34 (−4.31 to 4.98)	0.887	0.977	0.989
TCF7L2 rs7903146	Daily coffee × allele	Fat mass (kg)	458	0.54 (−5.02 to 6.09)	0.850	0.977	0.989
TCF7L2 rs7903146	Weekly coffee × allele	Body fat (%)	458	0.83 (−2.08 to 3.74)	0.575	0.731	0.946
TCF7L2 rs7903146	Daily coffee × allele	Body fat (%)	458	−0.74 (−4.21 to 2.74)	0.677	0.731	0.946
TCF7L2 rs7903146	Weekly coffee × allele	Fat-free mass (kg)	458	−1.18 (−4.00 to 1.64)	0.411	0.648	0.946
TCF7L2 rs7903146	Daily coffee × allele	Fat-free mass (kg)	458	0.41 (−2.96 to 3.79)	0.810	0.648	0.946
TCF7L2 rs7903146	Weekly coffee × allele	Muscle mass (kg)	331	−0.24 (−1.63 to 1.15)	0.736	0.752	0.946
TCF7L2 rs7903146	Daily coffee × allele	Muscle mass (kg)	331	0.49 (−1.18 to 2.15)	0.565	0.752	0.946
TCF7L2 rs7903146	Weekly coffee × allele	Waist circumference (cm)	460	−2.08 (−7.49 to 3.33)	0.450	0.688	0.946
TCF7L2 rs7903146	Daily coffee × allele	Waist circumference (cm)	460	0.78 (−5.65 to 7.21)	0.811	0.688	0.946
TCF7L2 rs7903146	Weekly coffee × allele	Hip circumference (cm)	460	−1.45 (−5.76 to 2.85)	0.508	0.529	0.946
TCF7L2 rs7903146	Daily coffee × allele	Hip circumference (cm)	460	1.93 (−3.19 to 7.05)	0.459	0.529	0.946

Variant	Interaction term	Outcome	n	β or OR (95% CI)	p (term)	p (joint test)	q (joint test)
TCF7L2 rs7903146	Weekly coffee × allele	Waist–hip ratio	460	−0.0088 (−0.0361 to 0.0185)	0.527	0.649	0.946
TCF7L2 rs7903146	Daily coffee × allele	Waist–hip ratio	460	−0.0133 (−0.0457 to 0.0191)	0.420	0.649	0.946
TCF7L2 rs7903146	Weekly coffee × allele	Visceral fat (%)	331	2.76 (−2.26 to 7.77)	0.280	0.074	0.710
TCF7L2 rs7903146	Daily coffee × allele	Visceral fat (%)	331	−5.20 (−11.20 to 0.80)	0.089	0.074	0.710
TCF7L2 rs7903146	Weekly coffee × allele	Fasting glucose (mg/dL)	425	2.44 (−1.72 to 6.59)	0.250	0.500	0.946
TCF7L2 rs7903146	Daily coffee × allele	Fasting glucose (mg/dL)	425	1.29 (−3.88 to 6.46)	0.623	0.500	0.946
TCF7L2 rs7903146	Weekly coffee × allele	2-h glucose (mg/dL)	423	0.79 (−11.15 to 12.74)	0.896	0.510	0.946
TCF7L2 rs7903146	Daily coffee × allele	2-h glucose (mg/dL)	423	8.73 (−6.13 to 23.60)	0.249	0.510	0.946
TCF7L2 rs7903146	Weekly coffee × allele	Glucose AUC (scaled 0–1)	415	−0.0081 (−0.0421 to 0.0259)	0.639	0.761	0.946
TCF7L2 rs7903146	Daily coffee × allele	Glucose AUC (scaled 0–1)	415	0.0095 (−0.0311 to 0.0501)	0.646	0.761	0.946
TCF7L2 rs7903146	Weekly coffee × allele	HbA1c (%)	425	0.12 (−0.05 to 0.29)	0.170	0.359	0.878
TCF7L2 rs7903146	Daily coffee × allele	HbA1c (%)	425	0.08 (−0.14 to 0.29)	0.485	0.359	0.878
TCF7L2 rs7903146	Weekly coffee × allele	Fasting insulin (μIU/mL)	425	−2.63 (−5.66 to 0.40)	0.088	0.227	0.725
TCF7L2 rs7903146	Daily coffee × allele	Fasting insulin (μIU/mL)	425	−0.26 (−4.03 to 3.52)	0.894	0.227	0.725
TCF7L2 rs7903146	Weekly coffee × allele	Insulin AUC (scaled 0–1)	412	−0.0143 (−0.0578 to 0.0292)	0.519	0.175	0.725
TCF7L2 rs7903146	Daily coffee × allele	Insulin AUC (scaled 0–1)	412	−0.0489 (−0.1008 to 0.0030)	0.065	0.175	0.725
TCF7L2 rs7903146	Weekly coffee × allele	HOMA-IR	425	−0.65 (−1.42 to 0.12)	0.099	0.214	0.725
TCF7L2 rs7903146	Daily coffee × allele	HOMA-IR	425	0.11 (−0.85 to 1.07)	0.816	0.214	0.725
TCF7L2 rs7903146	Weekly coffee × allele	HOMA-B	424	−25.31 (−86.39 to 35.77)	0.416	0.632	0.946
TCF7L2 rs7903146	Daily coffee × allele	HOMA-B	424	12.17 (−63.86 to 88.20)	0.753	0.632	0.946
TCF7L2 rs7903146	Weekly coffee × allele	Total cholesterol (mg/dL)	432	1.69 (−12.77 to 16.16)	0.818	0.746	0.946
TCF7L2 rs7903146	Daily coffee × allele	Total cholesterol (mg/dL)	432	6.71 (−10.58 to 23.99)	0.446	0.746	0.946
TCF7L2 rs7903146	Weekly coffee × allele	LDL cholesterol (mg/dL)	432	0.54 (−12.87 to 13.96)	0.936	0.988	0.989
TCF7L2 rs7903146	Daily coffee × allele	LDL cholesterol (mg/dL)	432	1.22 (−14.82 to 17.25)	0.881	0.988	0.989
TCF7L2 rs7903146	Weekly coffee × allele	HDL cholesterol (mg/dL)	432	1.51 (−3.39 to 6.41)	0.545	0.702	0.946
TCF7L2 rs7903146	Daily coffee × allele	HDL cholesterol (mg/dL)	432	2.10 (−3.76 to 7.95)	0.482	0.702	0.946
TCF7L2 rs7903146	Weekly coffee × allele	Triglycerides (mg/dL)	432	−1.80 (−25.35 to 21.75)	0.881	0.453	0.937
TCF7L2 rs7903146	Daily coffee × allele	Triglycerides (mg/dL)	432	16.97 (−11.17 to 45.11)	0.237	0.453	0.937
TCF7L2 rs7903146	Weekly coffee × allele	Prevalent T2DM	460 (32)	OR 0.76 (0.13 to 4.10)	0.744	0.153	0.710
TCF7L2 rs7903146	Daily coffee × allele	Prevalent T2DM	460 (32)	OR 0.20 (0.02 to 1.02)	0.081	0.153	0.710
TCF7L2 rs7903146	Frequent snacking × allele	Body weight (kg)	461	−2.93 (−8.65 to 2.79)	0.314	0.314	0.804
TCF7L2 rs7903146	Frequent snacking × allele	BMI (kg/m²)	461	−1.20 (−3.10 to 0.70)	0.216	0.216	0.725
TCF7L2 rs7903146	Frequent snacking × allele	Fat mass (kg)	459	−3.09 (−7.06 to 0.88)	0.126	0.126	0.710
TCF7L2 rs7903146	Frequent snacking × allele	Body fat (%)	459	−2.52 (−5.00 to −0.04)	0.046	0.046	0.710
TCF7L2 rs7903146	Frequent snacking × allele	Fat-free mass (kg)	459	0.38 (−2.05 to 2.80)	0.760	0.760	0.946
TCF7L2 rs7903146	Frequent snacking × allele	Muscle mass (kg)	332	0.10 (−1.10 to 1.29)	0.873	0.873	0.962
TCF7L2 rs7903146	Frequent snacking × allele	Waist circumference (cm)	461	−3.54 (−8.16 to 1.08)	0.133	0.133	0.710
TCF7L2 rs7903146	Frequent snacking × allele	Hip circumference (cm)	461	−0.17 (−3.87 to 3.53)	0.928	0.928	0.976

Variant	Interaction term	Outcome	n	β or OR (95% CI)	p (term)	p (joint test)	q (joint test)
TCF7L2 rs7903146	Frequent snacking × allele	Waist–hip ratio	461	−0.0312 (−0.0545 to −0.0080)	0.008	0.008	0.496
TCF7L2 rs7903146	Frequent snacking × allele	Visceral fat (%)	332	−3.78 (−8.12 to 0.57)	0.088	0.088	0.710
TCF7L2 rs7903146	Frequent snacking × allele	Fasting glucose (mg/dL)	426	−4.06 (−7.62 to −0.49)	0.026	0.026	0.594
TCF7L2 rs7903146	Frequent snacking × allele	2-h glucose (mg/dL)	424	−3.83 (−14.25 to 6.59)	0.471	0.471	0.945
TCF7L2 rs7903146	Frequent snacking × allele	Glucose AUC (scaled 0–1)	416	−0.0199 (−0.0487 to 0.0090)	0.176	0.176	0.725
TCF7L2 rs7903146	Frequent snacking × allele	HbA1c (%)	426	0.05 (−0.09 to 0.20)	0.480	0.480	0.946
TCF7L2 rs7903146	Frequent snacking × allele	Fasting insulin (μIU/mL)	426	−2.01 (−4.64 to 0.62)	0.134	0.134	0.710
TCF7L2 rs7903146	Frequent snacking × allele	Insulin AUC (scaled 0–1)	413	−0.0310 (−0.0680 to 0.0060)	0.101	0.101	0.710
TCF7L2 rs7903146	Frequent snacking × allele	HOMA-IR	426	−0.61 (−1.28 to 0.06)	0.075	0.075	0.710
TCF7L2 rs7903146	Frequent snacking × allele	HOMA-B	425	−1.02 (−53.68 to 51.64)	0.970	0.970	0.989
TCF7L2 rs7903146	Frequent snacking × allele	Total cholesterol (mg/dL)	433	−4.86 (−17.31 to 7.60)	0.444	0.444	0.928
TCF7L2 rs7903146	Frequent snacking × allele	LDL cholesterol (mg/dL)	433	−3.23 (−14.77 to 8.32)	0.583	0.583	0.946
TCF7L2 rs7903146	Frequent snacking × allele	HDL cholesterol (mg/dL)	433	3.61 (−0.60 to 7.82)	0.093	0.093	0.710
TCF7L2 rs7903146	Frequent snacking × allele	Triglycerides (mg/dL)	433	−26.18 (−46.28 to −6.09)	0.011	0.011	0.496
TCF7L2 rs7903146	Frequent snacking × allele	Prevalent T2DM	461 (32)	OR 1.71 (0.49 to 6.67)	0.412	0.406	0.890

Interaction terms (behaviour × number of minor alleles; additive genetic model) from models that also included the main effects of the behaviour and the variant and were adjusted for age, sex, daily energy intake, and physical inactivity. Minor alleles: BDNF rs10501087 C; TCF7L2 rs4506565 T, rs7901695 C, and rs7903146 T. Reference categories: never coffee consumption and rare snacking. Estimates are regression coefficients (β, in the units of the outcome) for continuous outcomes and odds ratios (OR) for prevalent T2DM; for T2DM, n is the number of participants with the number of cases in parentheses, and with 27–33 cases these estimates are imprecise. Glucose and insulin AUC are scaled 0–1, as in Table S1. p (term), p-value of the individual interaction coefficient; p (joint test), F test (likelihood-ratio test for T2DM) of all interaction terms for the exposure (for coffee, the weekly and daily terms jointly); q (joint test), Benjamini–Hochberg false discovery rate-adjusted p-value across all 184 joint tests. Eleven joint tests had $p < 0.05$ (about 9 expected by chance) and none had $q < 0.05$. AUC, area under the curve; BMI, body mass index; CI, confidence interval; HbA1c, glycated haemoglobin; HDL, high-density lipoprotein; HOMA-B and HOMA-IR, homeostatic model assessment of β-cell function and insulin resistance; LDL, low-density lipoprotein; T2DM, type 2 diabetes.

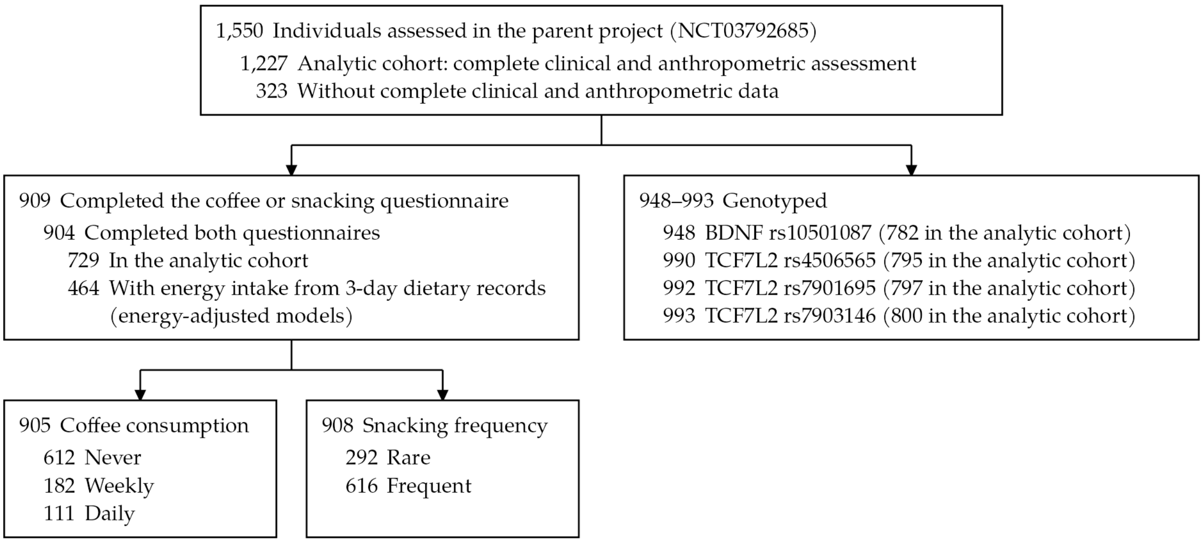

Figure S1. Participant flow diagram. Of the 1,550 individuals assessed in the parent project (NCT03792685), 1,227 had complete clinical and anthropometric assessment and formed the analytic cohort. Behavioural analyses included all participants who completed the coffee consumption (n = 905) or snacking frequency (n = 908) questionnaire; 904 completed both, of whom 729 were in the analytic cohort and 464 had energy intake from 3-day dietary records, which was used in the energy-adjusted models. Genotype analyses included all participants genotyped for each variant (n = 948–993; 782–800 in the analytic cohort). The coffee consumption and snacking frequency groups correspond to Tables 1 and 2, and the genotyped participants to Table 3.

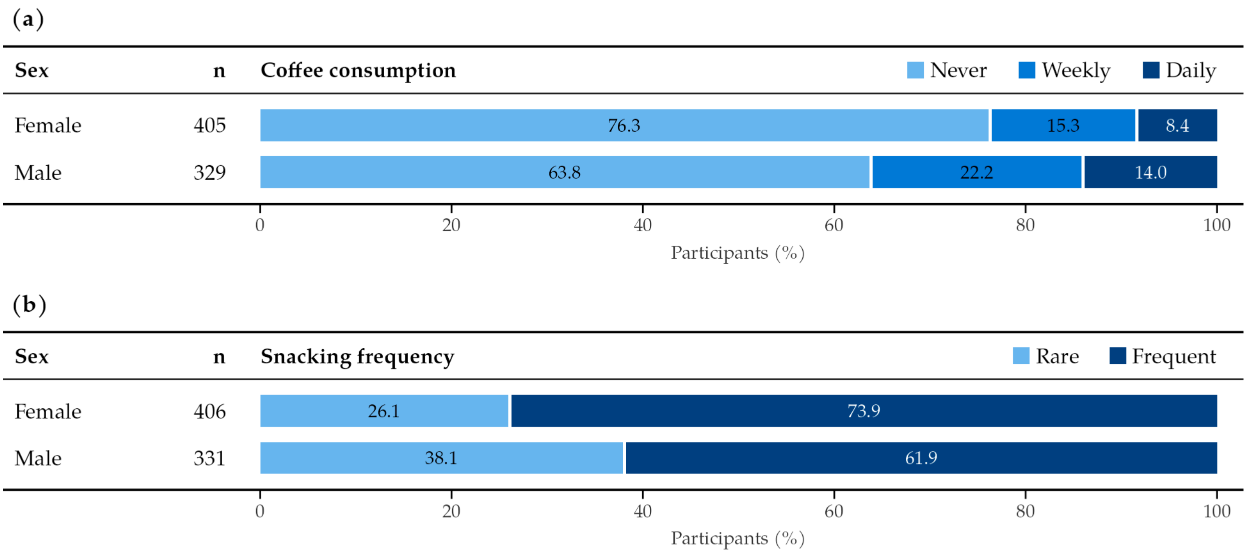

Figure S2. Patterns of coffee consumption and snacking by sex: (a) coffee consumption preferences between female and male participants; (b) frequency of snacking between female and male participants. Bars show, for each sex, the percentage of participants in each category; n, number of participants with data.

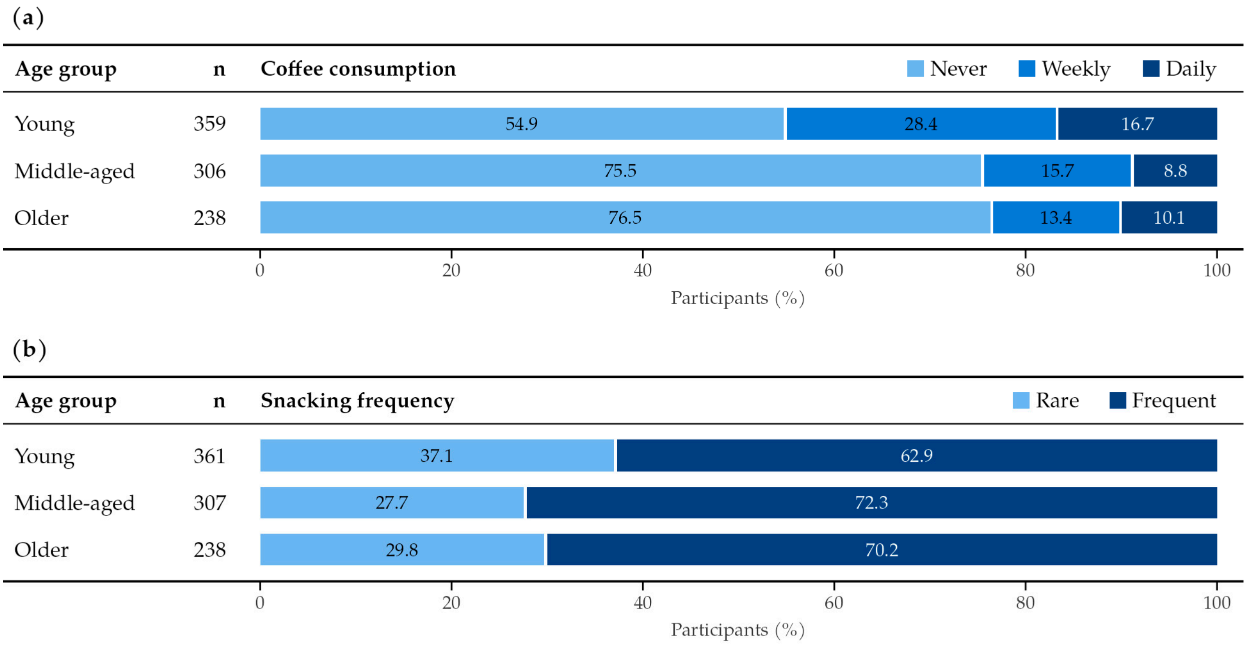

Figure S3. Distribution of coffee consumption and snacking habits by age group: (a) coffee consumption levels among young, middle-aged, and older participants; (b) frequency of snacking among young, middle-aged, and older participants. The participants were divided into three age categories: young (17.1–32.7 years), middle-aged (32.7–52.8 years), and older (52.8–79.0 years). Bars show, for each age group, the percentage of participants in each category; n, number of participants with data.
